# Monocyte derived dendritic cells generated by IFN-α acquire mature dendritic and natural killer cell properties as shown by gene expression analysis

**DOI:** 10.1186/1479-5876-5-46

**Published:** 2007-09-25

**Authors:** Mark Korthals, Nancy Safaian, Ralf Kronenwett, Dagmar Maihöfer, Matthias Schott, Claudia Papewalis, Elena Diaz Blanco, Meike Winter, Akos Czibere, Rainer Haas, Guido Kobbe, Roland Fenk

**Affiliations:** 1 Department of Hematology, Oncology and Clinical Immunology, Heinrich-Heine-University, Duesseldorf, Germany; 2 Department of Endocrinology, Diabetes and Rheumatology, Heinrich-Heine-University, Duesseldorf, Germany; 3 Institute for Transplantation Diagnostics and Cell Therapeutics, Heinrich-Heine-University, Duesseldorf, Germany; 4 Siemens Medical Solutions Diagnostics GmbH, Molecular Research Germany, Leverkusen, Germany

## Abstract

**Background:**

Dendritic cell (DC) vaccines can induce antitumor immune responses in patients with malignant diseases, while the most suitable DC culture conditions have not been established yet. In this study we compared monocyte derived human DC from conventional cultures containing GM-CSF and IL-4/TNF-α (IL-4/TNF-DC) with DC generated by the novel protocol using GM-CSF and IFN-α (IFN-DC).

**Methods:**

To characterise the molecular differences of both DC preparations, gene expression profiling was performed using Affymetrix microarrays. The data were conformed on a protein level by immunophenotyping, and functional tests for T cell stimulation, migration and cytolytic activity were performed.

**Results:**

Both methods resulted in CD11c+ CD86+ HLA-DR+ cells with a typical DC morphology that could efficiently stimulate T cells. But gene expression profiling revealed two distinct DC populations.

Whereas IL-4/TNF-DC showed a higher expression of genes envolved in phagocytosis IFN-DC had higher RNA levels for markers of DC maturity and migration to the lymph nodes like DCLAMP, CCR7 and CD49d. This different orientation of both DC populations was confined by a 2.3 fold greater migration in transwell experiments (p = 0.01).

Most interestingly, IFN-DC also showed higher RNA levels for markers of NK cells such as TRAIL, granzymes, KLRs and other NK cell receptors. On a protein level, intracytoplasmatic TRAIL and granzyme B were observed in 90% of IFN-DC. This translated into a cytolytic activity against K562 cells with a median specific lysis of 26% at high effector cell numbers as determined by propidium iodide uptake, whereas IL-4/TNF-DC did not induce any tumor cell lysis (p = 0.006). Thus, IFN-DC combined characteristics of mature DC and natural killer cells.

**Conclusion:**

Our results suggest that IFN-DC not only stimulate adaptive but also mediate innate antitumor immune responses. Therefore, IFN-DC should be evaluated in clinical vaccination trials. In particular, this could be relevant for patients with diseases responsive to a treatment with IFN-α such as Non-Hodgkin lymphoma or chronic myeloid leukemia.

## Background

Dendritic cells (DC) are specialized in antigen presentation which plays a key role in the initiation of primary immune responses. Immature DC phagocyte and process antigens and after maturation they stimulate antigen specific T cells. This is the prerequisite for orchestrating the cellular and humoral immune response [1].

This unique role of DC in the activation of host defense has made them a promising candidate for vaccination against a wide range of infectious agents and tumor antigens. DC can be generated by culturing monocytes *in vitro *with medium containing interleukin (IL)-4 and granulocyte-macrophage colony-stimulating factor (GM-CSF). TNF-α or a mixture of different proinflammatory molecules are needed to generate mature DC [2,3]. So far, the therapeutic results observed in patients with malignancies following vaccination with IL-4-DC are encouraging at best [4,5]. Therefore, there is a particular need for culture conditions facilitating the generation of more efficacious DC.

Recently, several groups generated DC by culturing monocytes in the presence of IFN-α and GM-CSF (IFN-DC) for three days [6-11]. IFN-α is released in large amounts during antiviral immune responses and is involved in the activation of cells of the innate and adaptive immune system [12]. In particular, IFN-α enhances the cytotoxic capacity of NK cells. IFN-α has also been successfully used for the treatment of patients with chronic myeloid leukemia (CML) [13] and Non-Hodgkin lymphoma (NHL) [14]. The therapeutical effects could be related to IFN-α stimulated NK cells and DC. Therefore, it is conceiving that IFN-DC would be more efficient for vaccination of patients with NHL or CML.

In order to examine the differences between IFN-DC and conventional IL-4/TNF-DC, we compared the morphology, immunophenotype, functional efficacy and gene expression profiles of these cell preparations with regard to their usefullness in anti-tumor vaccination strategies.

## Methods

### Isolation and culture of cells

Mononuclear cells (PBMC) were obtained from buffy coats of healthy individuals. Monocytes were isolated by negative selection using a RosetteSep antibody cocktail (Stemcell Technologies, Vancouver, Canada), according to the manufacturer's protocol. The resulting cell population after this procedure had a median purity of 72% CD14+ monocytes.

IFN-DC were generated by culturing monocytes in plastic flasks (BD Falcon, UK) for 3 days in serumfree X-VIVO 20 medium (BioWhitaker Europe, Belgium), supplemented with 1000 U/ml IFN-α (IntronA, Griffith Micro Science, Rantigny, France) and 1000 U/ml GM-CSF (Immunex, Seattle, US). For the generation of IL-4/TNF-DC, monocytes were cultured in serumfree medium containing 500 U/ml IL-4 (Promocell, Heidelberg, Germany) and 800 U/ml GM-CSF for 5 days. The resulting immature DC were further treated by a 2 day culture step with fresh medium containing 1000 U/ml TNF-α (Sigma) and 800 U/ml GM-CSF. For all experiments, IFN-DC and IL-4/TNF-DC were used after a culture period of 3 and 7 days, respectively. If not mentioned otherwise, preparations of both groups were derived from different individuals. The viability of cells was determined by Trypan blue exclusion.

### Immunophenotypic analysis

Flow cytometry was performed on a FACScan flow cytometer (BD Biosciences, San Jose, US). The following FITC or PE labeled mouse antibodies were used: CD45, CD1a, CD3, CD11c, CD14, CD19, CD40, CD49b, CD56, CD80, CD83, CD86, CD123, HLA-DR, TRAIL, NKG2D, nonspecific IgG1, IgG2a, a mixture of IgG1 and IgG2a (BD Biosciences, San Jose, US), CD209 (Beckman Coulter, Marseille, France) and GZMB (Hölzel Diagnostika, Köln, Germany). For intracellular staining, cells were permeabilized with BD Cytofix/Cytoperm (BD Bioscience, San Jose, US) according to the manufacturer's guideline.

### Analysis of DC functions

The allostimulatory capacity of DC was measured in an allogeneic mixed leukocyte reaction (MLR). DC were resuspended in RPMI 1640 medium (Biochrome, Berlin, Germany), supplemented with 10% FCS (PAA, Pasching, Austria), 2 mM Glutamine, 100 U/ml Penicillin and 100 μg/ml Streptomycin (Sigma) and irradiated with 30 Gy. Different DC numbers were cultivated with 1 × 10^5 ^allogeneic PBMC of a healthy donor in a round-bottomed 96-well plate (Corning, NY, US). Antibodies against CD28 and CD49d (BD Biosciences, San Jose, US) were added at 1 μg/ml. The cells were incubated for 4 days at 37°C. For the last 20 h of the culture, 1 μCi/well of ^3 ^[H]-Thymidin (Amersham, Braunschweig, Germany) was added. Finally, ^3 ^[H]-Thymidin uptake was measured on a β-scintillation counter (Perkin Elmer, Shelton, CT, US). The stimulatory capacity was expressed by the stimulation index SI = cpm of stimulated PBMC/cpm of unstimulated PBMC. Each experiment was done in triplicates.

Induction of cytokine production in T cells by DC was determined by intracellular staining. As described above, 1 × 10^5 ^freshly isolated PBMC were cocultured with 5 × 10^4 ^DC per well in a 96-well plate for 3 days. To block protein secretion, 10 μg/ml BrefeldinA (Sigma) was added for the last 4 hours of the culture period. Cells were harvested and incubated with a FITC labelled anti CD3 antibody (BD Bioscience, San Jose, US). Cells were then permeabilized with BD Cytofix/Cytoperm solution, stained with PE labelled anti IFN-γ or IL-4 antibodies (BD Biosciences, San Jose, US) and analyzed by flow cytometry.

Migration of DC was measured in 24-well transwell culture chambers (Costar, Cambridge, MA, US) as previously described [8,15]. The 8 μm-pore transwell filters were briefly coated with 10 μg/cm^2 ^fibronectin (Sigma). The upper chamber compartment was loaded with 2.5 × 10^5 ^IFN-DC or IL-4/TNF-DC in 150 μl X-VIVO 20 medium. The lower chamber compartment was filled with 500 μl medium supplemented with 100 ng/ml recombinant Mip-3β (Promocell, Heidelberg, Germany). After 2 h incubation at 37°C, cells were harvested from the lower chamber compartment and counted by FACS analysis using BD calibration beads.

### Cytotoxicity assay

Freshly prepared DC preparations derived from the same healthy individuals for both methods were tested for their cytolytic activity against K562 target cells by flow cytometry. Before coculture, 1 × 10^6 ^K562 cells were labeled with 0.5 μM carboxyfluorescein diacetate succinimidyl ester (CFDA-SE, Molecular probes, Paisley, UK) for 15 min. at 37°C. Different numbers of effector cells were cultured with 1 × 10^5 ^labelled K562 cells in RPMI 1640 medium containing 10% FCS. NK cells used as a positive control were isolated from peripheral blood of a healthy donor with a MACS NK cell separation kit (Miltenyi, Bergisch Gladbach, Germany), and stimulated with 1000 U/ml IL-2 (Chiron). The human lymphoma B cell line MHH-PREB-1 (German Collection of Microorganisms and Cell Cultures, Braunschweig, Germany) served as a negative control. After 4 h of culture dead cells were stained with propidium iodide (PI, Becton Dickinson) and analyzed by flow cytometry. Specific lysis was determined by the formula: % specific lysis = experimental % of PI+ CFDA-SE labeled cells - spontanous % of PI+ CFDA-SE labeled cells.

### Identification of differential gene expression

Total RNA from DC preparations was isolated with the RNeasy Mini kit (Qiagen, Hilden, Germany) according to the manufacturer's recommendations. cRNA labelling, hybridization to HG-Focus GeneChips (Affymetrix, UK, Ltd.) and processing of the microarray data was performed as described elsewhere [16]. Differential gene expression was defined by a *false discovery rate *(FDR) of 5%, as indicated by a q-value ≤ 5% for individual genes. To reduce the number of genes, we concentrated on genes with a fold change ≥ 2 comparing both groups. For a specific analysis of NK cell markers, all differentially expressed genes with q-values ≤ 5% were included.

### RT-PCR analysis

cDNA was synthesized from the same RNA preparations used for the microarray hybridizations as described [17], and amplified using the Assays-on-Demand Gene Expression products on the ABI PRISM 7900HT sequence detection system instrument (Applied Biosystems, Applera Deutschland GmbH, Darmstadt, Germany). For relative quantification, β2-microglobulin mRNA served as an external standard. The following genes were detected by Assays-on-Demand gene expression products: CCL8, BCL2A1, GZMB, CCR7, LAMP3, PKR, ADAMDEC1, FCGR1A, PDHA1, CCND2 and B2M. Relative gene expression levels were expressed as the difference in Ct values (deltaCt) of the target gene and B2M.

### Statistical analysis

The results were analysed for statistical significance by a two-sided, unpaired student's t-test for experiments with independent samples and a two-sided, paired student's t-test for paired samples. p-values < 0.05 indicate significant differences.

## Results

### IFN-DC and IL-4/TNF-DC show typical DC characteristics

#### Viability and morphology

The resulting DC populations (n = 34) for both culture conditions had a median purity of 84% as measured by FSC/SSC characteristics and the proportion of CD11c and HLA-DR positive cells. DC yield was equal with both methods with a median proportion of 25% of the initial monocyte count. As a result of the shorter culture period, IFN-DC had a significantly greater viability than IL-4/TNF-DC with median values of > 99% (n = 11) and 89% (n = 7), respectively (p = 0.003).

**Figure 1:**
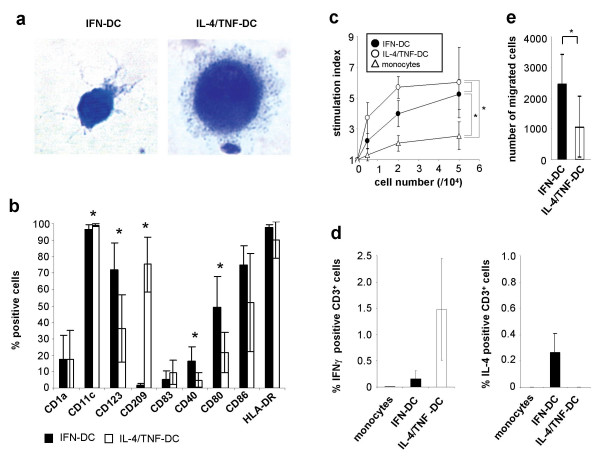
Phenotypical and functional characteristics of IFN-DC in comparison to IL-4/TNF-DC. (a) Morphology of IFN-DC (left) and IL-4/TNF-DC (right). Cells were stained according to Pappenheim and viewed at x630 magnification. (b) Immunophenotypic analysis of IFN-DC and IL-4/TNF-DC. DC were gated according to their FSC/SSC characteristics. The results are summarized as mean ± SD of % PE positive CD45+ cells. (c) Stimulatory potential of DC in an allogeneic MLR. Different numbers of DC or monocytes were cocultured with 1 × 10^5 ^PBMC for 4 days in the presence of anti CD28 and CD49d antibodies. ^3 ^[H]-Thymidin was added, and incorporated radioactivity was measured by β-scintillation. Results are expressed as the mean ± SD of the stimulation index. (d) Cytokine production of allogeneic T cells. Allogeneic PBMC were cocultured in a 2:1 ratio with monocytes, IFN-DC or IL-4/TNF-DC or without any stimulus for three days. IFN-γ (left) and IL-4 (right) expression was detected by intracellular flow cytometry. The results are summarized as mean ± SD of % cytokine positive CD3+ cells after subtraction of background levels. (e) Migratory capacity of DC. Migration was measured in 24-well transwell culture chambers. Each well was loaded with 2.5 × 10^5 ^cells. The proportion of migrated cells towards Mip-3β as measured by flow cytometry is summarized as mean ± SD. Significant differences (p < 0.05) are marked by an asterisk. The brackets and asteriscs in c refer to the highest effector cell number.

IFN-DC and IL-4/TNF-DC had a similar morphology typical for DC. On the other hand, IFN-DC were smaller, less granular and had a polarized distribution of cytoplasmic protrusions, whereas IL-4/TNF-DC showed uniformly distributed protrusions (Fig. 1a).

#### Immunophenotypic characterization

The immunophenotypic analysis of IFN-DC and IL-4/TNF-DC (n = 8 each) revealed a strong reduction of CD14 surface expression compared to monocytes (data not shown). Both DC populations had characteristic surface markers for DC, expressing CD11c, CD86 and HLA-DR (Fig. 1b). The proportion of cells positively staining for the costimulatory molecules CD40 and CD80, was greater among the population of IFN-DC than within the population of IL-4/TNF-DC (CD40: 14 ± 9% vs 4 ± 4%, p = 0.01 and CD80: 49 ± 19% vs 22 ± 13%, p = 0.006). The plasmacytoid DC marker CD123 was found on a significantly greater proportion of IFN-DC in comparison to IL-4/TNF-DC (72 ± 16% vs 36 ± 20%, p = 0.003), whereas CD209, an adhesion molecule known to be expressed exclusively on IL-4-DC, was not present on IFN-DC.

#### Allostimulatory capacity

IFN-DC (n = 4) as well as IL-4/TNF-DC (n = 4) induced proliferation of allogeneic PBMC in a dose dependent manner in an allogeneic MLR and stimulation was significantly higher with both DC populations in comparison to monocytes (n = 5) (Fig. 1c).

Intracellular staining of IFN-γ and IL-4 in allogeneic T cells after coculture with DC (Fig. 1d) revealed that both, IFN-DC (n = 3) and IL-4/TNF-DC (n = 3), but not monocytes (n = 2), could induce IFN-γ production in T cells. Moreover, IFN-DC but not IL-4/TNF-DC simultanously induced also IL-4 production.

### IFN-DC and IL-4/TNF-DC represent two distinct DC populations

**Figure 2:**
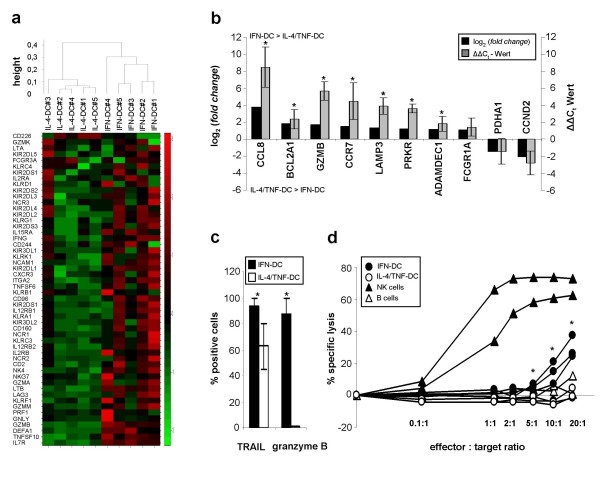
IFN-DC have novel molecular, phenotypical and functional characteristics in comparison to IL-4/TNF-DC. (a) Hierarchical cluster analysis of 52 genes related to NK cell function (rows) for IFN-DC and IL-4/TNF-DC preparations (columns) with expression levels obtained by Affymetrix microarray analysis. The colour scale indicates upregulation (green) and downregulation (red) of gene expression in relation to the mean expression levels of all preparations. (b) Corroboration of the microarray data by quantitative real-time PCR. The ΔCt values of 10 genes with β2-microglobulin as a reference gene were measured. Differences of the relative expression (mean ΔΔCt) of each gene were compared to the log2 of the fold change determined by microarray technology. (c) Expression of cytolytic effector molecules by DC. The intracellular expression of TRAIL and granzyme B by IFN-DC and IL-4/TNF-DC was analyzed by flow cytometry after permeabilization of cell membranes. The results are shown as mean ± SD of % positive cells. (d) Cytolytic activity of DC. Specific lysis of tumor cells by DC was measured by flow cytometric detection of propidium iodide uptake after coculture of 1 × 10^5 ^CFDA-SE labeled K562 cells with different numbers of IFN-DC and IL-4/TNF-DC as indicated by the effector : target ratios. IL-2 activated NK-cells were used as a positive, and B cells as negative control. Significant differences between IFN-DC and IL-4/TNF-DC at different effector : target ratios are indicated by an asterisk (p < 0.05).

In order to have a closer look at molecular differences between IFN-DC and IL-4/TNF-DC, gene expression profiles of five DC preparations for each culture condition were examined. We found 689 genes from a total of 8793 probe sets to be differentially expressed with a q-value < 5% and a fold change > 2. For corroboration, the differential expression of 10 genes detected by microarray technology was determined by quantitative real-time PCR for three IFN-DC and IL-4/TNF-DC preparations each. The more than twofold higher expression of 8 genes in IFN-DC and two genes in IL-4/TNF-DC could be verified in good accordance (Fig. 2b). Of the 689 differentially expressed genes, 131 had a more than twofold greater expression level in IFN-DC, while 558 genes were more than twofold higher expressed in IL-4/TNF-DC (Additional file 2 and 3). Hierarchical cluster analysis for these 689 genes (Additional file 1) showed that IFN-DC and IL-4/TNF-DC represent two distinct DC populations by a strict grouping of IFN-DC versus IL-4/TNF-DC. In the following, we describe the differentially expressed genes by assigning them to functional DC characteristics.

#### Signaling pathways of IFN-α, IL-4 and TNF-α

As expected, the cytokines used for the culture of IFN-DC and IL-4/TNF-DC activated signaling pathways of IFN-α or IL-4 and TNF-α, respectively. Transcription factors of the IFN pathway, like STAT1, IRF7 and ISGF3G, were expressed to a greater extent in IFN-DC as well as genes coding for antiproliferative and antiviral effector molecules like PKR, Mx1, oligoadenylate synthetases and other typical interferon stimulated genes [18,19].

On the other hand, greater expression levels of genes involved in IL-4 specific responses such as alterations of lipid metabolism, chemotaxis, adhesion and phagocytosis could be observed in IL-4/TNF-DC. These genes include for example ALOX15, MDC, CD209, and FCER2 [20]. Similarly, typical TNF-α induced genes involved in intracellular signaling and transcriptional regulation like NFKBIA and EGR1 were higher expressed in IL-4/TNF-DC  [21].

#### Differences in the maturation status

In order to assess the maturation status of both DC populations we had a look at genes associated with phagocytosis, antigen presentation and migration to the lymph nodes as well as genes for proinflammatory mediators for activation of immunocompetent cells. Looking at genes involved in phagocytosis, which is associated with a more immature DC phenotype, most of the differentially expressed genes had greater levels in IL-4/TNF-DC, like the genes for Ig receptors FCGRIIB, FCAR and FCER2, complement components and receptors C1QA, C3 and C1QR1, and the C-type lectins CD209 and CD205. IFN-DC had only higher levels of the IgG receptor FCGR1A.

In regard to antigen presentation, there were no differences in the expression levels of MHC molecules and other antigen presenting molecules. Some genes that participate in lysosomal antigen processing were differentially expressed. Of interest, the RNA level of the lysosomal associated membrane protein DCLAMP which is upregulated in mature DC [22], was higher in IFN-DC.

On the other hand, there were marked differences between IFN-DC and IL-4/TNF-DC in the expression of genes influencing migration and adhesion. IL-4/TNF-DC showed a greater expression of genes involved in adhesion to epithelium and inflammed tissue and T cell interaction like integrin αE chain, CD97, and the β2 integrins CD18, CD11b and CD11c. In contrast, IFN-DC had higher RNA levels of the chemokine receptor CCR7 and the integrin α4 chain, that play important roles in the migration of mature DC to the lymph nodes [23,24]. Besides, they also expressed higher RNA levels for molecules related to adhesion to lymphoid tissue like sialoadhesin and decysin [25,26], as well as the antiapoptotic protein bcl2A1 that mediates survival of naïve T cells and is also expressed by mature DC [27].

We confirmed this finding by functional analysis of the migratory capacity of both DC populations on functional level. IFN-DC (n = 4) showed a significantly 2.3 fold greater proportion (p = 0.01) of migrated cells towards the chemokine Mip-3β compared with IL-4/TNF-DC (n = 4) (Fig. 1e).

Finally, IFN-DC and IL-4/TNF-DC also differentially expressed proinflammatory mediators. IFN-DC had greater RNA levels for the chemokines MCP1, MCP2 and MCP3, potent attractors and activators of leukocytes [28], as well as MIP2A, MIP2B and PPBP which more specifically attract innate effector cells [29,30]. This indicates that IFN-DC may trigger a massive leukocyte migration. IFN-DC had also higher RNA levels for ISG15, IFN-β, IL-4 and IL-1β converting enzyme, which further potentiate IFN responses and contribute to the activation of NK cells as well as B cells and T cells [12,31,32]. IL-4/TNF-DC expressed a different set of chemokines, including MDC, RANTES and Mip-3β, which are attractors especially of activated T cells to inflammed tissues [28,33]. On the other hand, IL-4/TNF-DC produced greater amounts of RNA for enzymes of the lipid metabolism like ALOX5 and ALOX15, that catalyze the synthesis of proinflammatory leukotriens and other lipid metabolites, that are thought to play a role in DC differentiation [34].

#### Cytotoxic capacity

The most intriguing result of the gene expression analysis was the finding that IFN-DC expressed greater RNA levels of cytotoxic effector molecules, like the granzymes B and M, TRAIL and defensin-α1, which are important components of the cytotoxic arsenal of NK cells [35,36]. Therefore, we looked at 52 NK cell associated genes presented by the microarray. Cluster analysis demonstrated a distinct expression profile of these genes by IFN-DC in comparison to IL-4/TNF-DC (Fig. 2a). Of these genes, 32 were differentially expressed, all of them with significantly higher levels in IFN-DC than in IL-4/TNF-DC such as CD49b, KLRK1/NKG2D, NCR2/NKp44 and NCR1/NKp46 (Table 1).

**Table 1:** Genes involved in NK cell function with significantly higher expression in IFN-DC in comparison to IL-4/TNF-DC (q-value <5%)

Gene Symbol	Gene Symbol	Fold change
IL7R	interleukin 7 receptor	8.66
TRAIL	tumor necrosis factor (ligand) superfamily, member 10	5.02
DEFA1	defensin, alpha 1, myeloid-related sequence	4.64
GZMB	granzyme B (cytotoxic T-lymphocyte-associated serine esterase 1)	3.33
GZMM	granzyme M (lymphocyte met-ase 1)	2.09
KLRF1	killer cell lectin-like receptor subfamily F, member 1	1.91
LAG3	lymphocyte-activation gene 3	1.88
LTB	lymphotoxin beta (TNF superfamily, member 3)	1.83
GZMA	granzyme A (cytotoxic T-lymphocyte-associated serine esterase 3)	1.83
NKG7	natural killer cell group 7 sequence	1.77
CD2	CD2 antigen (p50), sheep red blood cell receptor	1.64
NCR2	natural cytotoxicity triggering receptor 2	1.55
IL2RB	interleukin 2 receptor, beta	1.53
IL12RB2	interleukin 12 receptor, beta 2	1.48
KLRC3	killer cell lectin-like receptor subfamily C, member 3	1.45
NCR1	natural cytotoxicity triggering receptor 1	1.45
NK1	CD160 antigen	1.42
KIR3DL2	killer cell immunoglobulin-like receptor, three domains, long cytoplasmic tail, 2	1.41
KLRA1	killer cell lectin-like receptor subfamily A, member 1	1.34
IL12RB1	interleukin 12 receptor, beta 1	1.33
KIR2DS1	killer cell immunoglobulin-like receptor, two domains, short cytoplasmic tail, 1	1.33
CD96	CD96 antigen	1.33
FasL	tumor necrosis factor (ligand) superfamily, member 6	1.32
CD49b	integrin, alpha 2 (CD49B, alpha 2 subunit of VLA-2 receptor)	1.30
CXCR3	chemokine (C-X-C motif) receptor 3	1.27
KIR2DL1	killer cell immunoglobulin-like receptor, two domains, long cytoplasmic tail, 1	1.27
CD56	neural cell adhesion molecule 1	1.27
KLRK1	killer cell lectin-like receptor subfamily K, member 1	1.27
IFNG	interferon, gamma	1.21
IL15RA	interleukin 15 receptor, alpha	1.21
KIR2DS3	killer cell immunoglobulin-like receptor, two domains, short cytoplasmic tail, 3	1.19
KLRG1	killer cell lectin-like receptor subfamily G, member 1	1.19

To corroborate these results, DC were further analyzed for their cytotoxic capacity on protein level. TRAIL expression (n = 6 for IFN-DC, n = 3 for IL-4/TNF-DC) was detected intracellularly in almost all IFN-DC and in a significantly lower proportion of IL-4/TNF-DC (94% vs 62%, p = 0.004) (Fig. 2c). In addition, the intracellular expression of granzyme B (n = 3 each) was solely found in IFN-DC (88% vs 1%, p < 0.001).

Further, these differences between IFN-DC and IL-4/TNF-DC could also be demonstrated on a functional level. We tested the cytotolytic activity of both DC preparations on K562 cells by measurement of propidium iodide uptake. Three DC preparations for each protocol were derived from the same three healthy individuals and tested in duplicates. IFN-DC displayed a significant dose dependent cytotoxicity on K562 cells in a 4 h coculture experiment as shown by a mean specific lysis of 26% at an effector : target cell ratio of 20 : 1 (p = 0.006), 15% at 10 : 1 (p = 0.03) and 5% at 5 : 1 (p = 0.04), whereas IL-4/TNF-DC did not show any specific cytotoxicity (Fig. 2d).

## Discussion

DC based vaccines for patients with malignant diseases generated under different culture conditions have been investigated for more than a decade. Despite these efforts, clinical results of DC vaccination studies showed therapeutic efficacy only in a limited number of patients so far [4]. In search of an alternative way for DC generation we examined the molecular and functional characteristics of IFN-DC in comparison to IL-4/TNF-DC.

We could show that both, IFN-DC and IL-4/TNF-DC, display typical DC characteristics, but also have distinct molecular and functional phenotypes, as a reflection of the distinct transcriptional signature of IFN-α in comparison to other cytokines as recently described [19]. Our results from gene expression analysis confirm previous reports that IFN-DC have signs of a pronounced maturation state and an increased migratory capacity to the lymph nodes in comparison to IL-4/TNF-DC [8,10]. Strikingly, IFN-DC showed a more plasmacytoid phenotype associated with NK cell characteristics on molecular and protein level as well as a functional cytotoxic activity against tumor cells. Therefore, the use of IFN-DC in vaccination trials may result in a better clinical antitumor immune response.

As others have shown before [6-11], IFN-DC in our study had a DC morphology and immunophenotype with high levels of CD11c, CD86 and HLA-DR as well as functional DC characteristics like the capacity to stimulate T cells. The expression of costimulatory molecules was in accordance to other studies using serumfree culture conditions [37]. Nevertheless, we found a stronger upregulation of the costimulatory molecules CD40 and CD80 on IFN-DC than on IL-4/TNF-DC, although IFN-DC did not mediate an increased allostimulatory reaction. Further, IFN-DC triggered a balanced Th1/Th2 response, whereas IL-4/TNF-DC were strongly biased to evoke a Th1 response. This is in line with Lapenta *et al*., 2003 and 2006, who showed that IFN-DC could induce a massive humoral and cellular immune response [9,38].

In addition, the greater RNA levels for cytokines and chemokines in IFN-DC like IFN-β, MCPs, MIP2A and MIP2B as well as the IL-1β converting enzyme (CASP1) that catalyzes the secretion of active forms of IL-1β and IL-18, suggest that IFN-DC may also recruit other innate cytotoxic effectors like NK cells [12,28,29,31,32] and neutrophils [29,30].

It is well accepted that a pronounced DC maturation status is important for the induction of efficient immune responses by DC immunotherapy [39]. In our study, both DC preparations showed only marginal upregulation of the maturation marker CD83, which is a result from culture conditions using serum free medium [37]. Gene expression analysis revealed that IL-4/TNF-DC have more immature DC characteristics. This was indicated by the higher expression of several genes envolved in phagocytosis such as Fc and complement receptors as well as genes envolved in epithelial adhesion structure formation including the genes for vinculin or the integrin αE chain. In contrast to this finding, IFN-DC showed a higher expression of several alternative DC maturation markers than IL-4/TNF-DC that are involved in antigen processing (DCLAMP [22]), migration to and localization in the lymph nodes (CCR7 [23], integrin α4 [24] and decysin [26]) as well as survival (BCL2A1 [27]). Therefore, on molecular level, IFN-DC show the prerequisite to initiate an adaptive immune response in the lymph node [1,39]. Importantly, this capacity of IFN-DC could be demonstrated functionally by a higher migratory capacity of IFN-DC *in vitro *compared to IL-4/TNF-DC as shown by transwell experiments. This is also in line with Parlato *et al*., 2001, who had shown before that IFN-DC have a higher migratory potency than IL-4-DC not stimulated by TNF-α [8].

Interestingly, the higher expression of genes of the IFN pathway like the transcription factors STAT1 and IRF7 as well as PKR, and IFN-β in IFN-DC resembles the expression pattern of plasmacytoid DC [40-43] that are the major type I IFN producers during viral infections [44]. We found high surface levels of the plasmacytoid DC marker CD123 and low levels of the myeloid DC marker CD209 on IFN-DC, which is in line with Mohty *et. al*., 2003, who also described other plasmacytoid DC markers like TLR7 on IFN-DC [45].

The most important new finding of our study was the significant upregulation of 32 genes strongly related to NK cell functions in IFN-DC compared to IL-4/TNF-DC. These include NK cell receptors NKp80, NKp44, NKp46 and NKG2D that are synergizing the cytotoxic activity of NK cells [46,47], as well as CD56 and cytotoxic effector molecules such as granzymes and TRAIL. Indeed, on protein level, we could detect intracellular pools of TRAIL and granzyme B in IFN-DC. Finally, as a further corroboration of the suggested cytotoxic capacity, IFN-DC but not IL-4/TNF-DC were able to kill K562 cells *in vitro*. This is in concordance with the previously made observation that DC can kill tumor cells by TRAIL mediated lysis [7,48]. Still, the expression of granzymes might further argue for a perforin mediated killing mechanism by IFN-DC.

These findings are of particular interest, as a new murine DC cell population has been recently described, termed interferon-producing killer dendritic cells (IKDC), that express molecular markers of plasmacytoid DC and NK cells [49,50]. IKDC exhibit specific cytolytic activity upon contact with tumor cells or activation with CpG oligonucleotides and subsequently upregulate costimulatory molecules, migrate to the lymph nodes and present antigen to T cells. Indeed, 9 of the genes specifically expressed by IKDC including granzymes, NKG2D, NKp46, and CD49b as detemined by microarray analsysis [49], were also differentially expressed by IFN-DC in comparison to IL-4/TNF-DC. Together with the pronounced migratory potential and the cytotoxic capacity of IFN-DC, the similarities between IFN-DC and mouse IKDC suggest that also in humans a molecular and functional relationship exists between DC and NK cells.

## Conclusion

In conclusion, the results of this study convey the idea, that IFN-α does not only induce DC differentiation. It further triggers maturation of a distinct DC type with NK cell properties that is not only capable of inducing a specific primary antitumor response in the lymph nodes but also mediates an innate immune response. IFN-DC can not only stimulate T cells but can kill tumor cells by themselves. Due to these new functional properties, IFN-DC are a promising alternative for vaccination strategies, that should be evaluated in clinical trials.

## Competing interests

The author(s) declare that they have no competing interests.

## Authors' contributions

MK, RK, RH, GK, RF designed the experiments.

MK, NS, DM, CP, EDB, MW, RF performed experiments and data analysis.

MK, RK, MS, RH, GK, RF performed interpretation of the data.

MK, RK, MS, AC, RH, GK, RF were involved in drafting the manuscript.

All authors were involved in revising the manuscript critically for important intellectual content and have given their approval to the final version of the manuscript.

## Supplementary Material

Additional file 1 Hierarchical cluster analysis of IFN-DC and IL-4/TNF-DC. The figure demonstrates that IFN-DC and IL-4/TNF-DC represent two distinct DC populations as seen by a strict grouping of 5 IFN-DC versus 5 IL-4/TNF-DC preparations in the hierarchical cluster analysis for 689 differentially expressed genes with a q-value < 5% and a fold change ≥ 2. Click here for file

Additional file 2 Complete list of genes higher expressed in IFN-DC with a fold change > 2 and a q-value < 5 % in comparison to IL-4/TNF-DC. The table includes the Affymetrix number, symbol and name of the 131 genes that are higher expressed in IFN-DC than in IL-4/DC and the corresponding fold change and q-value for each gene as determined by the SAM algorithm. Click here for file

Additional file 3 Complete list of genes higher expressed in IL-4/TNF-DC with a fold change > 2 and a q-value < 5 % in comparison to IFN-DC. The table includes the Affymetrix number, symbol and name of the 558 genes that are higher expressed in IL-4/TNF-DC and the corresponding fold change and q-value for each gene as determined by the SAM algorithm. Click here for file
