# Supplementary figures and images for: Monocyte derived dendritic cells generated by IFN-α acquire mature dendritic and natural killer cell properties as shown by gene expression analysis
Source: J Transl Med. 2007 Sep 25;5:46. doi: 10.1186/1479-5876-5-46 (PMC2064912; doi:10.1186/1479-5876-5-46)

## Slide 1
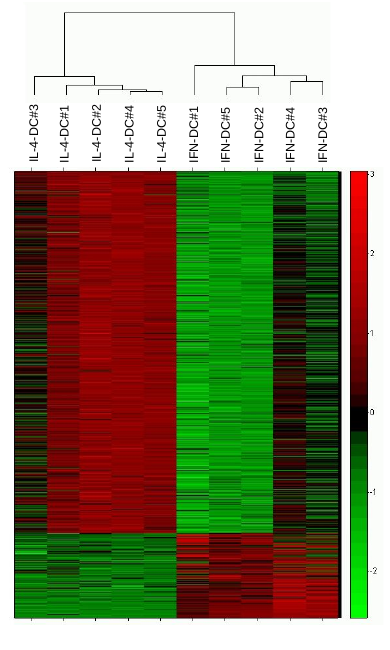

IL-4-DC#3
IL-4-DC#2
IL-4-DC#1
IL-4-DC#4
IL-4-DC#5
IFN-DC#1
IFN-DC#2
IFN-DC#5
IFN-DC#4
IFN-DC#3

Supplement: Additional file 1 — Hierarchical cluster analysis of IFN-DC and IL-4/TNF-DC. The figure demonstrates that IFN-DC and IL-4/TNF-DC represent two distinct DC populations as seen by a strict grouping of 5 IFN-DC versus 5 IL-4/TNF-DC preparations in the hierarchical cluster analysis for 689 differentially expressed genes with a q-value < 5% and a fold change ≥ 2. [file 1479-5876-5-46-S1.ppt]
