# Supplementary material for: Monocyte derived dendritic cells generated by IFN-α acquire mature dendritic and natural killer cell properties as shown by gene expression analysis
Source: J Transl Med. 2007 Sep 25;5:46. doi: 10.1186/1479-5876-5-46 (PMC2064912; doi:10.1186/1479-5876-5-46)
Supplement: Additional file 2 — Complete list of genes higher expressed in IFN-DC with a fold change > 2 and a q-value < 5 % in comparison to IL-4/TNF-DC. The table includes the Affymetrix number, symbol and name of the 131 genes that are higher expressed in IFN-DC than in IL-4/DC and the corresponding fold change and q-value for each gene as determined by the SAM algorithm. [file 1479-5876-5-46-S2.doc]

| **Additional file 2:** Complete list of genes higher expressed in IFN-DC with a fold change > 2 and a q-value < 5 % in comparison to IL-4 / TNF-DC | | | | |
| --- | --- | --- | --- | --- |
| **Affymetrix**  **Gene Name** | **Symbol** | **Name** | **Fold change** | **q-value (%)** |
| 202086_at | MX1 | myxovirus (influenza virus) resistance 1, interferon-inducible protein p78 (mouse) | 36,27 | 0,06 |
| 203153_at | IFIT1 | interferon-induced protein with tetratricopeptide repeats 1 | 33,18 | 0,06 |
| 205483_s_at | G1P2 | interferon, alpha-inducible protein (clone IFI-15K) | 27,82 | 0,06 |
| 212203_x_at | IFITM3 | interferon induced transmembrane protein 3 (1-8U) | 16,40 | 0,06 |
| 214038_at | CCL8 | chemokine (C-C motif) ligand 8 | 13,66 | 0,06 |
| 216598_s_at | CCL2 | chemokine (C-C motif) ligand 2 | 13,59 | 0,06 |
| 214022_s_at | IFITM1 | interferon induced transmembrane protein 1 (9-27) | 12,70 | 0,06 |
| 204747_at | IFIT4 | interferon-induced protein with tetratricopeptide repeats 4 | 11,63 | 0,06 |
| 202411_at | IFI27 | interferon, alpha-inducible protein 27 | 11,62 | 0,06 |
| 205552_s_at | OAS1 | 2',5'-oligoadenylate synthetase 1, 40/46kDa | 10,95 | 0,06 |
| 218400_at | OAS3 | 2'-5'-oligoadenylate synthetase 3, 100kDa | 9,17 | 0,06 |
| 201315_x_at | IFITM2 | interferon induced transmembrane protein 2 (1-8D) | 9,04 | 0,06 |
| 214146_s_at | PPBP | pro-platelet basic protein (chemokine (C-X-C motif) ligand 7) | 8,95 | 0,06 |
| 205798_at | IL7R | interleukin 7 receptor | 8,66 | 0,06 |
| 208436_s_at | IRF7 | interferon regulatory factor 7 | 8,34 | 0,06 |
| 204972_at | OAS2 | 2'-5'-oligoadenylate synthetase 2, 69/71kDa | 8,30 | 0,06 |
| 204994_at | MX2 | myxovirus (influenza virus) resistance 2 (mouse) | 8,04 | 0,06 |
| 219863_at | CEB1 | cyclin-E binding protein 1 | 7,94 | 0,06 |
| 202145_at | LY6E | lymphocyte antigen 6 complex, locus E | 7,66 | 0,06 |
| 214453_s_at | IFI44 | interferon-induced protein 44 | 7,57 | 0,06 |
| 204439_at | C1orf29 | chromosome 1 open reading frame 29 | 7,48 | 0,06 |
| 204415_at | G1P3 | interferon, alpha-inducible protein (clone IFI-6-16) | 7,38 | 0,06 |
| 210029_at | INDO | indoleamine-pyrrole 2,3 dioxygenase | 6,38 | 0,06 |
| 208075_s_at | CCL7 | chemokine (C-C motif) ligand 7 /// chemokine (C-C motif) ligand 7 | 6,30 | 0,06 |
| 202446_s_at | PLSCR1 | phospholipid scramblase 1 | 5,48 | 0,06 |
| 202688_at | TNFSF10 | tumor necrosis factor (ligand) superfamily, member 10 | 5,02 | 0,06 |
| 203645_s_at | CD163 | CD163 antigen | 4,98 | 0,06 |
| 219211_at | USP18 | ubiquitin specific protease 18 | 4,98 | 0,06 |
| 206207_at | CLC | Charot-Leyden crystal protein | 4,78 | 1,97 |
| 202087_s_at | CTSL | cathepsin L | 4,71 | 0,06 |
| 205033_s_at | DEFA1 | defensin, alpha 1, myeloid-related sequence | 4,64 | 0,06 |
| 209774_x_at | CXCL2 | chemokine (C-X-C motif) ligand 2 | 4,51 | 0,06 |
| 33304_at | ISG20 | interferon stimulated gene 20kDa | 4,25 | 0,06 |
| 207741_x_at | TPSB2 | tryptase beta 2 | 4,21 | 2,43 |
| 206978_at | CCR2 | chemokine (C-C motif) receptor 2 | 4,12 | 0,70 |
| 203559_s_at | ABP1 | amiloride binding protein 1 (amine oxidase (copper-containing)) | 3,87 | 0,06 |
| 200923_at | LGALS3BP | lectin, galactoside-binding, soluble, 3 binding protein | 3,86 | 0,06 |
| 210797_s_at | OASL | 2'-5'-oligoadenylate synthetase-like /// 2'-5'-oligoadenylate synthetase-like | 3,76 | 0,06 |
| 218943_s_at | RIG-I | DEAD/H (Asp-Glu-Ala-Asp/His) box polypeptide | 3,75 | 0,06 |
| 206214_at | PLA2G7 | phospholipase A2, group VII (platelet-activating factor acetylhydrolase, plasma) | 3,72 | 0,06 |
| 205683_x_at | TPSB2 | tryptase beta 2 | 3,65 | 1,97 |
| 205681_at | BCL2A1 | BCL2-related protein A1 | 3,55 | 0,06 |
| 200986_at | SERPING1 | serine (or cysteine) proteinase inhibitor, clade G (C1 inhibitor), member 1, (angioedema, hereditary) | 3,55 | 0,06 |
| 210164_at | GZMB | granzyme B (granzyme 2, cytotoxic T-lymphocyte-associated serine esterase 1) | 3,33 | 0,06 |
| 210139_s_at | PMP22 | peripheral myelin protein 22 | 3,06 | 0,06 |
| 208012_x_at | SP110 | SP110 nuclear body protein | 3,02 | 0,06 |
| 209458_x_at | HBA2 | hemoglobin, alpha 2 | 2,95 | 2,17 |
| 207850_at | CXCL3 | chemokine (C-X-C motif) ligand 3 | 2,91 | 0,17 |
| 206337_at | CCR7 | chemokine (C-C motif) receptor 7 | 2,89 | 1,80 |
| 205237_at | FCN1 | ficolin (collagen/fibrinogen domain containing) 1 | 2,84 | 0,46 |

| 44673_at | SN | sialoadhesin | 2,83 | 0,06 |
| --- | --- | --- | --- | --- |
| 209906_at | C3AR1 | complement component 3a receptor 1 | 2,82 | 0,06 |
| 203595_s_at | IFIT5 | interferon-induced protein with tetratricopeptide repeats 5 | 2,81 | 0,70 |
| 208304_at | CCR3 | chemokine (C-C motif) receptor 3 | 2,80 | 1,80 |
| 200887_s_at | STAT1 | signal transducer and activator of transcription 1, 91kDa | 2,75 | 0,39 |
| 204787_at | Z39IG | Ig superfamily protein | 2,73 | 0,06 |
| 219209_at | MDA5 | melanoma differentiation associated protein-5 | 2,71 | 1,46 |
| 203473_at | SLCO2B1 | solute carrier organic anion transporter family, member 2B1 | 2,68 | 0,14 |
| 217979_at | TM4SF13 | transmembrane 4 superfamily member 13 | 2,67 | 0,14 |
| 205119_s_at | FPR1 | formyl peptide receptor 1 | 2,65 | 0,14 |
| 219684_at | IFRG28 | 28kD interferon responsive protein | 2,64 | 0,06 |
| 206133_at | HSXIAPAF1 | XIAP associated factor-1 | 2,62 | 0,06 |
| 204638_at | ACP5 | acid phosphatase 5, tartrate resistant | 2,60 | 0,70 |
| 201650_at | KRT19 | keratin 19 /// keratin 19 | 2,59 | 1,80 |
| 204187_at | GMPR | guanosine monophosphate reductase | 2,59 | 0,06 |
| 204534_at | VTN | vitronectin (serum spreading factor, somatomedin B, complement S-protein) | 2,58 | 0,06 |
| 200766_at | CTSD | cathepsin D (lysosomal aspartyl protease) | 2,55 | 0,70 |
| 209230_s_at | P8 | p8 protein (candidate of metastasis 1) | 2,55 | 0,06 |
| 209761_s_at | SP110 | SP110 nuclear body protein | 2,54 | 0,26 |
| 205569_at | LAMP3 | lysosomal-associated membrane protein 3 | 2,54 | 3,83 |
| 215071_s_at | --- | --- | 2,52 | 1,86 |
| 206746_at | BFSP1 | beaded filament structural protein 1, filensin | 2,48 | 1,80 |
| 217232_x_at | --- | --- | 2,47 | 3,83 |
| 220273_at | IL17B | interleukin 17B | 2,45 | 1,46 |
| 219896_at | CALCYON | calcyon protein | 2,44 | 1,25 |
| 208477_at | KCNC1 | potassium voltage-gated channel, Shaw-related subfamily, member 1 | 2,44 | 1,86 |
| 203979_at | CYP27A1 | cytochrome P450, family 27, subfamily A, polypeptide 1 | 2,44 | 0,10 |
| 208579_x_at | H2BFS | H2B histone family, member S | 2,44 | 0,98 |
| 204211_x_at | PRKR | protein kinase, interferon-inducible double stranded RNA dependent | 2,39 | 0,06 |
| 208582_s_at | DUX1 | double homeobox, 1 | 2,37 | 1,80 |
| 207067_s_at | HDC | histidine decarboxylase | 2,35 | 1,86 |
| 203882_at | ISGF3G | interferon-stimulated transcription factor 3, gamma 48kDa | 2,33 | 0,39 |
| 210523_at | BMPR1B | bone morphogenetic protein receptor, type IB | 2,32 | 1,25 |
| 219529_at | CLIC3 | chloride intracellular channel 3 | 2,29 | 0,26 |
| 221198_at | SCT | secretin | 2,28 | 1,80 |
| 211506_s_at | --- | --- | 2,27 | 2,17 |
| 202270_at | GBP1 | guanylate binding protein 1, interferon-inducible, 67kDa | 2,27 | 1,66 |
| 209911_x_at | HIST1H2BD | histone 1, H2bd | 2,27 | 1,80 |
| 204858_s_at | ECGF1 | endothelial cell growth factor 1 (platelet-derived) | 2,26 | 1,86 |
| 204881_s_at | UGCG | UDP-glucose ceramide glucosyltransferase | 2,26 | 0,48 |
| 206134_at | ADAMDEC1 | ADAM-like, decysin 1 | 2,24 | 1,97 |
| 214414_x_at | HBA2 | hemoglobin, alpha 2 | 2,24 | 2,17 |
| 205910_s_at | CEL | carboxyl ester lipase (bile salt-stimulated lipase) | 2,23 | 1,97 |
| 212224_at | ALDH1A1 | aldehyde dehydrogenase 1 family, member A1 | 2,20 | 0,06 |
| 221601_s_at | TOSO | regulator of Fas-induced apoptosis | 2,20 | 0,06 |
| 205715_at | BST1 | bone marrow stromal cell antigen 1 | 2,19 | 0,06 |
| 219552_at | POLYDOM | likely ortholog of mouse polydom /// likely ortholog of mouse polydom | 2,19 | 1,97 |
| 208554_at | POU4F3 | POU domain, class 4, transcription factor 3 | 2,16 | 1,80 |
| 205713_s_at | COMP | cartilage oligomeric matrix protein /// cartilage oligomeric matrix protein | 2,15 | 1,86 |
| 216950_s_at | FCGR1A | Fc fragment of IgG, high affinity Ia, receptor for (CD64) | 2,15 | 0,26 |
| 204275_at | SOLH | small optic lobes homolog (Drosophila) | 2,14 | 1,46 |
| 209500_x_at | TNFSF13 | tumor necrosis factor (ligand) superfamily, member 13 | 2,12 | 0,48 |
| 204802_at | RRAD | Ras-related associated with diabetes | 2,11 | 1,86 |
| 203964_at | NMI | N-myc (and STAT) interactor | 2,11 | 1,66 |
| 218231_at | NAGK | N-acetylglucosamine kinase | 2,10 | 1,80 |

| 221026_s_at | SCRT1 | scratch homolog 1, zinc finger protein (Drosophila) /// scratch homolog 1, zinc finger protein (Drosophila) | 2,10 | 2,43 |
| --- | --- | --- | --- | --- |
| 218559_s_at | MAFB | v-maf musculoaponeurotic fibrosarcoma oncogene homolog B (avian) | 2,09 | 0,48 |
| 207460_at | GZMM | granzyme M (lymphocyte met-ase 1) | 2,09 | 1,25 |
| 221463_at | CCL24 | chemokine (C-C motif) ligand 24 | 2,09 | 1,80 |
| 207373_at | HOXD10 | homeo box D10 | 2,09 | 1,66 |
| 207736_s_at | TNP2 | transition protein 2 (during histone to protamine replacement) | 2,08 | 1,66 |
| 204798_at | MYB | v-myb myeloblastosis viral oncogene homolog (avian) | 2,07 | 0,46 |
| 209806_at | HIST1H2BK | histone 1, H2bk | 2,07 | 2,17 |
| 208592_s_at | CD1E | CD1E antigen, e polypeptide /// CD1E antigen, e polypeptide | 2,06 | 2,17 |
| 205241_at | SCO2 | SCO cytochrome oxidase deficient homolog 2 (yeast) | 2,06 | 0,26 |
| 207352_s_at | GABRB2 | gamma-aminobutyric acid (GABA) A receptor, beta 2 | 2,06 | 1,80 |
| 208567_s_at | KCNJ12 | potassium inwardly-rectifying channel, subfamily J, member 12 /// potassium inwardly-rectifying channel, subfamily J, member 12 | 2,05 | 2,43 |
| 203650_at | PROCR | protein C receptor, endothelial (EPCR) | 2,05 | 0,06 |
| 204224_s_at | GCH1 | GTP cyclohydrolase 1 (dopa-responsive dystonia) | 2,05 | 0,48 |
| 214477_at | MLLT1 | myeloid/lymphoid or mixed-lineage leukemia (trithorax homolog, Drosophila); translocated to, 1 | 2,04 | 1,97 |
| 208173_at | IFNB1 | interferon, beta 1, fibroblast | 2,03 | 1,25 |
| 205954_at | RXRG | retinoid X receptor, gamma | 2,03 | 1,46 |
| 209496_at | RARRES2 | retinoic acid receptor responder (tazarotene induced) 2 | 2,03 | 2,43 |
| 207539_s_at | IL4 | interleukin 4 | 2,03 | 0,26 |
| 209351_at | KRT14 | keratin 14 (epidermolysis bullosa simplex, Dowling-Meara, Koebner) | 2,02 | 1,25 |
| 208546_x_at | HIST1H2BH | histone 1, H2bh | 2,02 | 0,14 |
| 211367_s_at | CASP1 | caspase 1, apoptosis-related cysteine protease (interleukin 1, beta, convertase) | 2,01 | 0,98 |
| 208239_at | --- | --- | 2,01 | 3,83 |
| 207935_s_at | KRT13 | keratin 13 | 2,00 | 1,25 |
| 213416_at | ITGA4 | integrin, alpha 4 (antigen CD49D, alpha 4 subunit of VLA-4 receptor) | 2,00 | 0,32 |
| 220136_s_at | CRYBA2 | crystallin, beta A2 | 2,00 | 2,43 |
