# Supplementary material for: Monocyte derived dendritic cells generated by IFN-α acquire mature dendritic and natural killer cell properties as shown by gene expression analysis
Source: J Transl Med. 2007 Sep 25;5:46. doi: 10.1186/1479-5876-5-46 (PMC2064912; doi:10.1186/1479-5876-5-46)
Supplement: Additional file 3 — Complete list of genes higher expressed in IL-4/TNF-DC with a fold change > 2 and a q-value < 5 % in comparison to IFN-DC. The table includes the Affymetrix number, symbol and name of the 558 genes that are higher expressed in IL-4/TNF-DC and the corresponding fold change and q-value for each gene as determined by the SAM algorithm. [file 1479-5876-5-46-S3.doc]

| **Additional file 3:** Complete list of genes higher expressed in IL-4 / TNF-DC with a fold change > 2 and a q-value < 5 % in comparison to IFN-DC | | | | |
| --- | --- | --- | --- | --- |
| **Affymetrix Gene Name** | **Symbol** | **Name** | **Fold change** | **q-value (%)** |
| 207328_at | ALOX15 | arachidonate 15-lipoxygenase | 19,16 | 0,06 |
| 207861_at | CCL22 | chemokine (C-C motif) ligand 22 | 10,87 | 0,06 |
| 208002_s_at | BACH | brain acyl-CoA hydrolase | 9,49 | 0,06 |
| 219434_at | TREM1 | triggering receptor expressed on myeloid cells 1 | 9,27 | 0,06 |
| 202627_s_at | SERPINE1 | serine (or cysteine) proteinase inhibitor, clade E (nexin, plasminogen activator inhibitor type 1), member 1 | 8,86 | 0,06 |
| 207277_at | CD209 | CD209 antigen | 7,64 | 0,06 |
| 218718_at | PDGFC | platelet derived growth factor C | 6,77 | 0,06 |
| 203549_s_at | LPL | lipoprotein lipase | 6,54 | 0,06 |
| 202499_s_at | SLC2A3 | solute carrier family 2 (facilitated glucose transporter), member 3 | 6,53 | 0,06 |
| 209146_at | SC4MOL | sterol-C4-methyl oxidase-like | 6,44 | 0,06 |
| 213164_at | MRPS6 | mitochondrial ribosomal protein S6 | 6,42 | 0,06 |
| 201920_at | SLC20A1 | solute carrier family 20 (phosphate transporter), member 1 | 6,05 | 0,06 |
| 201042_at | --- | --- | 5,61 | 0,06 |
| 202364_at | MXI1 | MAX interacting protein 1 | 5,41 | 0,06 |
| 201348_at | GPX3 | glutathione peroxidase 3 (plasma) | 5,40 | 0,06 |
| 202468_s_at | CTNNAL1 | catenin (cadherin-associated protein), alpha-like 1 | 5,34 | 0,06 |
| 201037_at | PFKP | phosphofructokinase, platelet | 5,23 | 0,06 |
| 204655_at | CCL5 | chemokine (C-C motif) ligand 5 | 5,03 | 0,06 |
| 218236_s_at | PRKCN | protein kinase C, nu | 4,98 | 0,06 |
| 209301_at | CA2 | carbonic anhydrase II | 4,86 | 0,06 |
| 213524_s_at | G0S2 | putative lymphocyte G0/G1 switch gene | 4,77 | 0,06 |
| 205990_s_at | WNT5A | wingless-type MMTV integration site family, member 5A | 4,70 | 0,06 |
| 218232_at | C1QA | complement component 1, q subcomponent, alpha polypeptide | 4,69 | 0,06 |
| 205676_at | CYP27B1 | cytochrome P450, family 27, subfamily B, polypeptide 1 | 4,68 | 0,06 |
| 205786_s_at | ITGAM | integrin, alpha M (complement component receptor 3, alpha; also known as CD11b (p170), macrophage antigen alpha polypeptide) | 4,64 | 0,06 |
| 36711_at | MAFF | v-maf musculoaponeurotic fibrosarcoma oncogene homolog F (avian) | 4,56 | 0,06 |
| 202878_s_at | C1QR1 | complement component 1, q subcomponent, receptor 1 | 4,55 | 0,06 |
| 221841_s_at | KLF4 | Kruppel-like factor 4 (gut) | 4,49 | 0,06 |
| 201030_x_at | LDHB | lactate dehydrogenase B | 4,45 | 0,06 |
| 34210_at | CDW52 | CDW52 antigen (CAMPATH-1 antigen) | 4,44 | 0,06 |
| 201998_at | SIAT1 | sialyltransferase 1 (beta-galactoside alpha-2,6-sialyltransferase) | 4,42 | 0,06 |
| 202286_s_at | TACSTD2 | tumor-associated calcium signal transducer 2 | 4,40 | 0,46 |
| 204326_x_at | MT1X | metallothionein 1X /// metallothionein 1X | 4,38 | 0,06 |
| 205055_at | ITGAE | integrin, alpha E (antigen CD103, human mucosal lymphocyte antigen 1; alpha polypeptide) | 4,37 | 0,06 |
| 203140_at | BCL6 | B-cell CLL/lymphoma 6 (zinc finger protein 51) | 4,31 | 0,06 |
| 203305_at | F13A1 | coagulation factor XIII, A1 polypeptide | 4,28 | 2,17 |
| 209267_s_at | SLC39A8 | solute carrier family 39 (zinc transporter), member 8 | 4,28 | 0,06 |
| 210184_at | ITGAX | integrin, alpha X (antigen CD11C (p150), alpha polypeptide) | 4,27 | 0,06 |
| 201625_s_at | INSIG1 | insulin induced gene 1 | 4,26 | 0,06 |
| 205081_at | CRIP1 | cysteine-rich protein 1 (intestinal) | 4,24 | 0,06 |
| 200978_at | MDH1 | malate dehydrogenase 1, NAD (soluble) | 4,24 | 0,06 |
| 218507_at | HIG2 | hypoxia-inducible protein 2 | 4,24 | 0,06 |
| 202888_s_at | ANPEP | alanyl (membrane) aminopeptidase (aminopeptidase N, aminopeptidase M, microsomal aminopeptidase, CD13, p150) | 4,21 | 0,06 |
| 200953_s_at | CCND2 | cyclin D2 | 4,13 | 0,06 |
| 205630_at | CRH | corticotropin releasing hormone | 4,04 | 0,06 |
| 201242_s_at | ATP1B1 | ATPase, Na+/K+ transporting, beta 1 polypeptide | 3,96 | 0,06 |
| 212271_at | MAPK1 | mitogen-activated protein kinase 1 | 3,88 | 0,06 |
| 204034_at | ETHE1 | ethylmalonic encephalopathy 1 | 3,88 | 0,06 |
| 203232_s_at | SCA1 | spinocerebellar ataxia 1 (olivopontocerebellar ataxia 1, autosomal dominant, ataxin 1) | 3,87 | 0,06 |
| 200705_s_at | EEF1B2 | eukaryotic translation elongation factor 1 beta 2 | 3,79 | 0,06 |
| 210512_s_at | VEGF | vascular endothelial growth factor | 3,78 | 0,06 |
| 200662_s_at | TOMM20 | translocase of outer mitochondrial membrane 20 homolog (yeast) | 3,76 | 0,06 |
| 206414_s_at | DDEF2 | development and differentiation enhancing factor 2 | 3,69 | 0,06 |
| 219938_s_at | PSTPIP2 | proline-serine-threonine phosphatase interacting protein 2 | 3,69 | 0,06 |
| 222062_at | WSX1 | class I cytokine receptor | 3,63 | 0,06 |
| 208662_s_at | TTC3 | tetratricopeptide repeat domain 3 | 3,59 | 0,06 |
| 204580_at | MMP12 | matrix metalloproteinase 12 (macrophage elastase) | 3,58 | 0,06 |
| 200972_at | TM4SF8 | transmembrane 4 superfamily member 8 | 3,57 | 0,06 |
| 217869_at | HSD17B12 | hydroxysteroid (17-beta) dehydrogenase 12 | 3,57 | 0,06 |
| 220091_at | SLC2A6 | solute carrier family 2 (facilitated glucose transporter), member 6 | 3,56 | 0,06 |
| 201989_s_at | CREBL2 | cAMP responsive element binding protein-like 2 | 3,51 | 0,06 |
| 39248_at | AQP3 | aquaporin 3 | 3,50 | 0,06 |
| 208841_s_at | G3BP2 | Ras-GTPase activating protein SH3 domain-binding protein 2 | 3,49 | 0,06 |
| 214709_s_at | KTN1 | kinectin 1 (kinesin receptor) | 3,43 | 0,06 |
| 217757_at | A2M | alpha-2-macroglobulin | 3,43 | 0,06 |
| 201029_s_at | CD99 | CD99 antigen | 3,43 | 0,06 |
| 200850_s_at | AHCYL1 | S-adenosylhomocysteine hydrolase-like 1 | 3,43 | 0,06 |
| 210950_s_at | FDFT1 | farnesyl-diphosphate farnesyltransferase 1 | 3,43 | 0,06 |
| 206022_at | NDP | Norrie disease (pseudoglioma) | 3,42 | 0,06 |
| 219939_s_at | D1S155E | NRAS-related gene | 3,41 | 0,06 |
| 217767_at | C3 | complement component 3 | 3,39 | 0,06 |
| 209118_s_at | TUBA3 | tubulin, alpha 3 | 3,38 | 0,06 |
| 208581_x_at | MT1X | metallothionein 1X | 3,35 | 0,10 |
| 209716_at | CSF1 | colony stimulating factor 1 (macrophage) /// colony stimulating factor 1 (macrophage) | 3,33 | 0,06 |
| 200832_s_at | SCD | stearoyl-CoA desaturase (delta-9-desaturase) | 3,31 | 0,17 |
| 210715_s_at | SPINT2 | serine protease inhibitor, Kunitz type, 2 | 3,27 | 0,06 |
| 214167_s_at | --- | Homo sapiens transcribed sequence with moderate similarity to protein sp:P05388 (H.sapiens) RLA0_HUMAN 60S acidic ribosomal protein P0 (L10E) | 3,26 | 0,06 |
| 221476_s_at | RPL15 | ribosomal protein L15 | 3,22 | 0,06 |
| 217846_at | QARS | glutaminyl-tRNA synthetase | 3,22 | 0,06 |
| 201432_at | CAT | catalase | 3,21 | 0,06 |
| 201560_at | CLIC4 | chloride intracellular channel 4 | 3,16 | 0,06 |
| 204388_s_at | MAOA | monoamine oxidase A | 3,15 | 0,06 |
| 207071_s_at | ACO1 | aconitase 1, soluble | 3,15 | 0,06 |
| 217967_s_at | C1orf24 | chromosome 1 open reading frame 24 | 3,14 | 0,10 |
| 201473_at | JUNB | jun B proto-oncogene | 3,13 | 0,06 |
| 211937_at | EIF4B | eukaryotic translation initiation factor 4B | 3,13 | 0,06 |
| 210889_s_at | FCGR2B | Fc fragment of IgG, low affinity IIb, receptor for (CD32) | 3,13 | 0,06 |
| 202539_s_at | HMGCR | 3-hydroxy-3-methylglutaryl-Coenzyme A reductase | 3,12 | 0,06 |
| 200931_s_at | VCL | vinculin | 3,10 | 0,06 |
| 201801_s_at | SLC29A1 | solute carrier family 29 (nucleoside transporters), member 1 | 3,10 | 0,06 |
| 204446_s_at | ALOX5 | arachidonate 5-lipoxygenase | 3,10 | 0,06 |
| 201502_s_at | NFKBIA | nuclear factor of kappa light polypeptide gene enhancer in B-cells inhibitor, alpha | 3,08 | 0,06 |
| 212600_s_at | UQCRC2 | ubiquinol-cytochrome c reductase core protein II | 3,06 | 0,06 |
| 201300_s_at | PRNP | prion protein (p27-30) (Creutzfeld-Jakob disease, Gerstmann-Strausler-Scheinker syndrome, fatal familial insomnia) | 3,04 | 0,06 |
| 212191_x_at | RPL13 | ribosomal protein L13 | 3,03 | 0,06 |
| 209218_at | SQLE | squalene epoxidase | 3,02 | 0,70 |
| 201972_at | ATP6V1A | ATPase, H+ transporting, lysosomal 70kDa, V1 subunit A | 3,01 | 0,06 |
| 201362_at | IVNS1ABP | influenza virus NS1A binding protein | 2,99 | 0,06 |
| 202912_at | ADM | adrenomedullin | 2,99 | 0,61 |
| 208161_s_at | ABCC3 | ATP-binding cassette, sub-family C (CFTR/MRP), member 3 | 2,98 | 0,06 |
| 209696_at | FBP1 | fructose-1,6-bisphosphatase 1 | 2,98 | 0,06 |
| 217871_s_at | MIF | macrophage migration inhibitory factor (glycosylation-inhibiting factor) | 2,97 | 0,06 |
| 216733_s_at | GATM | glycine amidinotransferase (L-arginine:glycine amidinotransferase) | 2,97 | 0,06 |
| 203680_at | PRKAR2B | protein kinase, cAMP-dependent, regulatory, type II, beta | 2,96 | 0,06 |
| 208697_s_at | EIF3S6 | eukaryotic translation initiation factor 3, subunit 6 48kDa | 2,95 | 0,06 |
| 206760_s_at | FCER2 | Fc fragment of IgE, low affinity II, receptor for (CD23A) | 2,95 | 0,06 |
| 203284_s_at | HS2ST1 | heparan sulfate 2-O-sulfotransferase 1 | 2,93 | 0,06 |
| 201903_at | UQCRC1 | ubiquinol-cytochrome c reductase core protein I | 2,93 | 0,06 |
| 212426_s_at | YWHAQ | tyrosine 3-monooxygenase/tryptophan 5-monooxygenase activation protein, theta polypeptide | 2,91 | 0,06 |
| 201022_s_at | DSTN | destrin (actin depolymerizing factor) | 2,91 | 0,06 |
| 209154_at | TIP-1 | Tax interaction protein 1 | 2,91 | 0,06 |
| 208714_at | NDUFV1 | NADH dehydrogenase (ubiquinone) flavoprotein 1, 51kDa | 2,90 | 0,06 |
| 203835_at | GARP | glycoprotein A repetitions predominant | 2,90 | 0,06 |
| 201170_s_at | BHLHB2 | basic helix-loop-helix domain containing, class B, 2 | 2,89 | 0,06 |
| 218036_x_at | CGI-07 | CGI-07 protein | 2,88 | 0,06 |
| 221266_s_at | DCSTAMP | DC-specific transmembrane protein /// DC-specific transmembrane protein | 2,88 | 0,06 |
| 207543_s_at | P4HA1 | procollagen-proline, 2-oxoglutarate 4-dioxygenase (proline 4-hydroxylase), alpha polypeptide I | 2,88 | 0,06 |
| 205105_at | MAN2A1 | mannosidase, alpha, class 2A, member 1 | 2,87 | 0,06 |
| 211023_at | PDHB | pyruvate dehydrogenase (lipoamide) beta | 2,87 | 0,06 |
| 208788_at | ELOVL5 | ELOVL family member 5, elongation of long chain fatty acids (FEN1/Elo2, SUR4/Elo3-like, yeast) | 2,86 | 0,10 |
| 200858_s_at | RPS8 | ribosomal protein S8 | 2,85 | 0,06 |
| 215157_x_at | PABPC1 | poly(A) binding protein, cytoplasmic 1 | 2,84 | 0,06 |
| 212099_at | ARHB | ras homolog gene family, member B | 2,84 | 0,06 |
| 201482_at | QSCN6 | quiescin Q6 | 2,84 | 0,06 |
| 221748_s_at | TNS | tensin | 2,84 | 0,06 |
| 201599_at | OAT | ornithine aminotransferase (gyrate atrophy) | 2,82 | 0,17 |
| 217870_s_at | UMP-CMPK | UMP-CMP kinase | 2,82 | 0,06 |
| 204057_at | ICSBP1 | interferon consensus sequence binding protein 1 | 2,82 | 0,06 |
| 207419_s_at | RAC2 | ras-related C3 botulinum toxin substrate 2 (rho family, small GTP binding protein Rac2) | 2,82 | 0,10 |
| 200823_x_at | RPL29 | ribosomal protein L29 | 2,82 | 0,06 |
| 201661_s_at | FACL3 | fatty-acid-Coenzyme A ligase, long-chain 3 | 2,82 | 0,39 |
| 218498_s_at | ERO1L | ERO1-like (S. cerevisiae) | 2,81 | 0,06 |
| 200761_s_at | JWA | cytoskeleton related vitamin A responsive protein | 2,80 | 0,06 |
| 200602_at | APP | amyloid beta (A4) precursor protein (protease nexin-II, Alzheimer disease) | 2,78 | 0,06 |
| 204490_s_at | CD44 | CD44 antigen (homing function and Indian blood group system) | 2,78 | 0,06 |
| 212181_s_at | NUDT4 | nudix (nucleoside diphosphate linked moiety X)-type motif 4 | 2,78 | 0,06 |
| 201847_at | LIPA | lipase A, lysosomal acid, cholesterol esterase (Wolman disease) | 2,77 | 0,06 |
| 208717_at | OXA1L | oxidase (cytochrome c) assembly 1-like | 2,77 | 0,06 |
| 217807_s_at | GLTSCR2 | glioma tumor suppressor candidate region gene 2 | 2,77 | 0,06 |
| 205698_s_at | MAP2K6 | mitogen-activated protein kinase kinase 6 | 2,77 | 0,06 |
| 200036_s_at | RPL10A | ribosomal protein L10a | 2,76 | 0,06 |
| 201064_s_at | PABPC4 | poly(A) binding protein, cytoplasmic 4 (inducible form) | 2,76 | 0,06 |
| 211564_s_at | RIL | LIM domain protein | 2,75 | 0,10 |
| 202218_s_at | FADS2 | fatty acid desaturase 2 | 2,75 | 0,06 |
| 207196_s_at | TNIP1 | TNFAIP3 interacting protein 1 | 2,75 | 0,06 |
| 202930_s_at | SUCLA2 | succinate-CoA ligase, ADP-forming, beta subunit | 2,74 | 0,17 |
| 201258_at | RPS16 | ribosomal protein S16 | 2,72 | 0,06 |
| 202362_at | RAP1A | RAP1A, member of RAS oncogene family | 2,72 | 0,06 |
| 203621_at | NDUFB5 | NADH dehydrogenase (ubiquinone) 1 beta subcomplex, 5, 16kDa | 2,72 | 0,06 |
| 208796_s_at | CCNG1 | cyclin G1 | 2,72 | 0,06 |
| 202646_s_at | D1S155E | NRAS-related gene | 2,71 | 0,06 |
| 200081_s_at | RPS6 | ribosomal protein S6 | 2,71 | 0,06 |
| 218285_s_at | DHRS6 | dehydrogenase/reductase (SDR family) member 6 | 2,71 | 0,06 |
| 214665_s_at | --- | Homo sapiens cDNA FLJ20088 fis, clone COL03869 | 2,70 | 0,06 |
| 200937_s_at | RPL5 | ribosomal protein L5 | 2,70 | 0,06 |
| 212360_at | AMPD2 | adenosine monophosphate deaminase 2 (isoform L) | 2,70 | 0,06 |
| 200094_s_at | --- | Homo sapiens transcribed sequence with strong similarity to protein sp:P13639 (H.sapiens) EF2_HUMAN Elongation factor 2 (EF-2) | 2,69 | 0,06 |
| 206461_x_at | MT1H | metallothionein 1H | 2,69 | 0,26 |
| 217860_at | NDUFA10 | NADH dehydrogenase (ubiquinone) 1 alpha subcomplex, 10, 42kDa | 2,69 | 0,06 |
| 204031_s_at | DKFZP564J157 | DKFZp564J157 protein | 2,69 | 0,06 |
| 215813_s_at | PTGS1 | prostaglandin-endoperoxide synthase 1 (prostaglandin G/H synthase and cyclooxygenase) | 2,68 | 0,39 |
| 202164_s_at | CNOT8 | CCR4-NOT transcription complex, subunit 8 | 2,68 | 0,06 |
| 200980_s_at | PDHA1 | pyruvate dehydrogenase (lipoamide) alpha 1 | 2,68 | 0,06 |
| 201160_s_at | CSDA | cold shock domain protein A | 2,67 | 0,06 |
| 200038_s_at | RPL17 | ribosomal protein L17 | 2,66 | 0,06 |
| 212320_at | OK/SW-cl.56 | beta 5-tubulin | 2,65 | 0,06 |
| 201772_at | OAZIN | ornithine decarboxylase antizyme inhibitor | 2,64 | 0,06 |
| 203547_at | CD4 | CD4 antigen (p55) | 2,64 | 0,06 |
| 213746_s_at | FLNA | filamin A, alpha (actin binding protein 280) | 2,64 | 0,14 |
| 200992_at | IPO7 | importin 7 | 2,64 | 0,10 |
| 212185_x_at | MT2A | metallothionein 2A | 2,62 | 0,26 |
| 201968_s_at | PGM1 | phosphoglucomutase 1 | 2,62 | 0,06 |
| 213846_at | COX7C | cytochrome c oxidase subunit VIIc | 2,62 | 0,06 |
| 201798_s_at | FER1L3 | fer-1-like 3, myoferlin (C. elegans) | 2,61 | 0,06 |
| 201272_at | AKR1B1 | aldo-keto reductase family 1, member B1 (aldose reductase) | 2,61 | 0,06 |
| 202467_s_at | TRIP15 | thyroid receptor interacting protein 15 | 2,61 | 0,06 |
| 203887_s_at | THBD | thrombomodulin | 2,61 | 0,06 |
| 202783_at | NNT | nicotinamide nucleotide transhydrogenase | 2,60 | 0,06 |
| 210511_s_at | INHBA | inhibin, beta A (activin A, activin AB alpha polypeptide) | 2,60 | 0,06 |
| 208666_s_at | ST13 | suppression of tumorigenicity 13 (colon carcinoma) (Hsp70 interacting protein) | 2,59 | 0,06 |
| 212688_at | PIK3CB | phosphoinositide-3-kinase, catalytic, beta polypeptide | 2,59 | 0,06 |
| 200804_at | TEGT | testis enhanced gene transcript (BAX inhibitor 1) | 2,59 | 0,06 |
| 200657_at | SLC25A5 | solute carrier family 25 (mitochondrial carrier; adenine nucleotide translocator), member 5 | 2,58 | 0,06 |
| 202381_at | ADAM9 | a disintegrin and metalloproteinase domain 9 (meltrin gamma) | 2,58 | 0,17 |
| 205936_s_at | HK3 | hexokinase 3 (white cell) | 2,58 | 0,06 |
| 203574_at | NFIL3 | nuclear factor, interleukin 3 regulated | 2,58 | 0,06 |
| 203710_at | ITPR1 | inositol 1,4,5-triphosphate receptor, type 1 | 2,57 | 0,26 |
| 201909_at | RPS4Y | ribosomal protein S4, Y-linked | 2,57 | 0,06 |
| 213129_s_at | --- | Homo sapiens transcribed sequence with strong similarity to protein pir:GCHUH (H.sapiens) GCHUH glycine cleavage system protein H precursor - human | 2,57 | 0,10 |
| 222021_x_at | --- | Homo sapiens similar to Succinate dehydrogenase [ubiquinone] flavoprotein subunit, mitochondrial precursor (Fp) (Flavoprotein subunit of complex II) (LOC375386), mRNA | 2,56 | 0,06 |
| 200099_s_at | --- | --- | 2,56 | 0,06 |
| 207992_s_at | AMPD3 | adenosine monophosphate deaminase (isoform E) | 2,56 | 0,06 |
| 213093_at | PRKCA | protein kinase C, alpha | 2,56 | 0,06 |
| 219549_s_at | RTN3 | reticulon 3 | 2,55 | 0,10 |
| 200023_s_at | EIF3S5 | eukaryotic translation initiation factor 3, subunit 5 epsilon, 47kDa | 2,55 | 0,06 |
| 221654_s_at | USP3 | ubiquitin specific protease 3 | 2,55 | 0,06 |
| 201812_s_at | TOMM7 | translocase of outer mitochondrial membrane 7 homolog (yeast) | 2,54 | 0,06 |
| 201007_at | HADHB | hydroxyacyl-Coenzyme A dehydrogenase/3-ketoacyl-Coenzyme A thiolase/enoyl-Coenzyme A hydratase (trifunctional protein), beta subunit | 2,54 | 0,06 |
| 200788_s_at | PEA15 | phosphoprotein enriched in astrocytes 15 | 2,54 | 0,06 |
| 202393_s_at | TIEG | TGFB inducible early growth response | 2,54 | 0,06 |
| 39729_at | PRDX2 | peroxiredoxin 2 | 2,54 | 0,26 |
| 200074_s_at | RPL14 | ribosomal protein L14 | 2,53 | 0,06 |
| 213457_at | MFHAS1 | malignant fibrous histiocytoma amplified sequence 1 | 2,53 | 0,06 |
| 200691_s_at | HSPA9B | heat shock 70kDa protein 9B (mortalin-2) | 2,52 | 0,17 |
| 204971_at | CSTA | cystatin A (stefin A) | 2,52 | 0,06 |
| 201600_at | REA | repressor of estrogen receptor activity | 2,52 | 0,06 |
| 201406_at | RPL36A | ribosomal protein L36a | 2,51 | 0,06 |
| 200809_x_at | RPL12 | ribosomal protein L12 | 2,51 | 0,06 |
| 209875_s_at | SPP1 | secreted phosphoprotein 1 (osteopontin, bone sialoprotein I, early T-lymphocyte activation 1) | 2,51 | 0,26 |
| 200614_at | CLTC | clathrin, heavy polypeptide (Hc) | 2,51 | 0,10 |
| 201707_at | PXF | peroxisomal farnesylated protein | 2,50 | 0,06 |
| 201389_at | ITGA5 | integrin, alpha 5 (fibronectin receptor, alpha polypeptide) | 2,50 | 0,06 |
| 202644_s_at | TNFAIP3 | tumor necrosis factor, alpha-induced protein 3 | 2,50 | 0,06 |
| 202829_s_at | SYBL1 | synaptobrevin-like 1 | 2,50 | 0,06 |
| 202429_s_at | PPP3CA | protein phosphatase 3 (formerly 2B), catalytic subunit, alpha isoform (calcineurin A alpha) | 2,50 | 0,46 |
| 201941_at | CPD | carboxypeptidase D | 2,49 | 0,06 |
| 202241_at | C8FW | phosphoprotein regulated by mitogenic pathways | 2,48 | 0,06 |
| 201339_s_at | SCP2 | sterol carrier protein 2 | 2,48 | 0,10 |
| 201666_at | TIMP1 | tissue inhibitor of metalloproteinase 1 (erythroid potentiating activity, collagenase inhibitor) | 2,47 | 0,06 |
| 203186_s_at | S100A4 | S100 calcium binding protein A4 (calcium protein, calvasculin, metastasin, murine placental homolog) | 2,47 | 0,14 |
| 202778_s_at | ZNF198 | zinc finger protein 198 | 2,47 | 0,06 |
| 217751_at | LOC51064 | glutathione S-transferase subunit 13 homolog | 2,47 | 0,06 |
| 200834_s_at | RPS21 | ribosomal protein S21 | 2,47 | 0,06 |
| 202233_s_at | UQCRH | ubiquinol-cytochrome c reductase hinge protein | 2,46 | 0,06 |
| 201290_at | SPC18 | signal peptidase complex (18kD) | 2,46 | 0,06 |
| 209448_at | HTATIP2 | HIV-1 Tat interactive protein 2, 30kDa | 2,46 | 0,06 |
| 211474_s_at | --- | --- | 2,46 | 0,06 |
| 217740_x_at | RPL7A | ribosomal protein L7a | 2,46 | 0,06 |
| 200089_s_at | RPL4 | ribosomal protein L4 | 2,45 | 0,06 |
| 202232_s_at | GA17 | dendritic cell protein | 2,44 | 0,10 |
| 219505_at | CECR1 | cat eye syndrome chromosome region, candidate 1 | 2,44 | 0,06 |
| 204093_at | CCNH | cyclin H | 2,44 | 0,06 |
| 200781_s_at | RPS15A | ribosomal protein S15a | 2,44 | 0,06 |
| 209444_at | RAP1GDS1 | RAP1, GTP-GDP dissociation stimulator 1 | 2,44 | 0,14 |
| 202605_at | GUSB | glucuronidase, beta | 2,43 | 0,06 |
| 204044_at | QPRT | quinolinate phosphoribosyltransferase (nicotinate-nucleotide pyrophosphorylase (carboxylating)) | 2,43 | 0,06 |
| 203156_at | AKAP11 | A kinase (PRKA) anchor protein 11 | 2,43 | 0,20 |
| 221743_at | CUGBP1 | CUG triplet repeat, RNA binding protein 1 | 2,43 | 0,10 |
| 200704_at | LITAF | lipopolysaccharide-induced TNF factor | 2,42 | 0,06 |
| 201236_s_at | BTG2 | BTG family, member 2 | 2,42 | 0,06 |
| 200862_at | DHCR24 | 24-dehydrocholesterol reductase | 2,42 | 0,70 |
| 208113_x_at | PABPC3 | poly(A) binding protein, cytoplasmic 3 /// poly(A) binding protein, cytoplasmic 3 | 2,42 | 0,06 |
| 211429_s_at | --- | Homo sapiens PRO2275 mRNA, complete cds | 2,42 | 0,46 |
| 200762_at | DPYSL2 | dihydropyrimidinase-like 2 | 2,42 | 0,32 |
| 221840_at | PTPRE | protein tyrosine phosphatase, receptor type, E | 2,41 | 1,66 |
| 202614_at | SLC30A9 | solute carrier family 30 (zinc transporter), member 9 | 2,41 | 0,06 |
| 219892_at | TM6SF1 | transmembrane 6 superfamily member 1 | 2,41 | 0,17 |
| 209036_s_at | MDH2 | malate dehydrogenase 2, NAD (mitochondrial) | 2,41 | 0,06 |
| 208754_s_at | NAP1L1 | nucleosome assembly protein 1-like 1 | 2,41 | 0,06 |
| 211423_s_at | SC5DL | sterol-C5-desaturase (ERG3 delta-5-desaturase homolog, fungal)-like | 2,41 | 0,14 |
| 202948_at | IL1R1 | interleukin 1 receptor, type I | 2,40 | 0,06 |
| 200088_x_at | --- | --- | 2,40 | 0,06 |
| 201417_at | SOX4 | SRY (sex determining region Y)-box 4 | 2,39 | 0,10 |
| 208655_at | CCNI | cyclin I | 2,39 | 0,10 |
| 212461_at | OAZIN | ornithine decarboxylase antizyme inhibitor | 2,39 | 0,06 |
| 212506_at | PICALM | phosphatidylinositol binding clathrin assembly protein | 2,38 | 0,06 |
| 201760_s_at | WSB2 | WD repeat and SOCS box containing protein 2 | 2,38 | 0,20 |
| 201268_at | NME2 | non-metastatic cells 2, protein (NM23B) expressed in | 2,38 | 0,06 |
| 200013_at | RPL24 | ribosomal protein L24 | 2,38 | 0,06 |
| 210549_s_at | CCL23 | chemokine (C-C motif) ligand 23 | 2,38 | 0,06 |
| 202085_at | TJP2 | tight junction protein 2 (zona occludens 2) | 2,38 | 0,06 |
| 209224_s_at | NDUFA2 | NADH dehydrogenase (ubiquinone) 1 alpha subcomplex, 2, 8kDa | 2,38 | 0,06 |
| 209903_s_at | ATR | ataxia telangiectasia and Rad3 related | 2,37 | 0,06 |
| 200947_s_at | GLUD1 | glutamate dehydrogenase 1 | 2,37 | 0,06 |
| 200726_at | PPP1CC | protein phosphatase 1, catalytic subunit, gamma isoform | 2,37 | 0,06 |
| 201892_s_at | IMPDH2 | IMP (inosine monophosphate) dehydrogenase 2 | 2,37 | 0,06 |
| 203336_s_at | ITGB1BP1 | integrin beta 1 binding protein 1 | 2,37 | 0,06 |
| 209884_s_at | SLC4A7 | solute carrier family 4, sodium bicarbonate cotransporter, member 7 | 2,37 | 0,06 |
| 200735_x_at | NACA | nascent-polypeptide-associated complex alpha polypeptide | 2,37 | 0,06 |
| 204103_at | CCL4 | chemokine (C-C motif) ligand 4 | 2,37 | 0,26 |
| 221523_s_at | RRAGD | Ras-related GTP binding D | 2,37 | 0,06 |
| 209248_at | GHITM | growth hormone inducible transmembrane protein | 2,37 | 0,10 |
| 205404_at | HSD11B1 | hydroxysteroid (11-beta) dehydrogenase 1 | 2,37 | 0,06 |
| 208764_s_at | ATP5G2 | ATP synthase, H+ transporting, mitochondrial F0 complex, subunit c (subunit 9), isoform 2 | 2,37 | 0,06 |
| 204912_at | IL10RA | interleukin 10 receptor, alpha | 2,37 | 0,06 |
| 208964_s_at | FADS1 | fatty acid desaturase 1 | 2,36 | 0,84 |
| 202990_at | PYGL | phosphorylase, glycogen; liver (Hers disease, glycogen storage disease type VI) | 2,36 | 0,06 |
| 219607_s_at | MS4A4A | membrane-spanning 4-domains, subfamily A, member 4 | 2,36 | 0,39 |
| 218024_at | BRP44L | brain protein 44-like | 2,35 | 0,06 |
| 200632_s_at | NDRG1 | N-myc downstream regulated gene 1 | 2,35 | 0,10 |
| 208121_s_at | PTPRO | protein tyrosine phosphatase, receptor type, O /// protein tyrosine phosphatase, receptor type, O | 2,34 | 0,61 |
| 212449_s_at | LYPLA1 | lysophospholipase I | 2,34 | 0,26 |
| 207198_s_at | LIMS1 | LIM and senescent cell antigen-like domains 1 | 2,34 | 0,39 |
| 201522_x_at | SNRPN | small nuclear ribonucleoprotein polypeptide N | 2,34 | 0,06 |
| 202217_at | C21orf33 | chromosome 21 open reading frame 33 | 2,34 | 0,06 |
| 203501_at | PGCP | plasma glutamate carboxypeptidase /// plasma glutamate carboxypeptidase | 2,34 | 0,06 |
| 218847_at | IMP-2 | IGF-II mRNA-binding protein 2 | 2,33 | 0,06 |
| 37012_at | CAPZB | capping protein (actin filament) muscle Z-line, beta | 2,33 | 0,06 |
| 205220_at | HM74 | putative chemokine receptor | 2,33 | 0,61 |
| 200717_x_at | RPL7 | ribosomal protein L7 | 2,33 | 0,06 |
| 201642_at | IFNGR2 | interferon gamma receptor 2 (interferon gamma transducer 1) | 2,33 | 0,06 |
| 205639_at | AOAH | acyloxyacyl hydrolase (neutrophil) | 2,33 | 0,10 |
| 208636_at | ACTN1 | actinin, alpha 1 | 2,33 | 0,26 |
| 208837_at | P24B | integral type I protein | 2,33 | 0,06 |
| 208611_s_at | SPTAN1 | spectrin, alpha, non-erythrocytic 1 (alpha-fodrin) | 2,32 | 0,06 |
| 209157_at | DNAJA2 | DnaJ (Hsp40) homolog, subfamily A, member 2 | 2,32 | 0,06 |
| 212586_at | CAST | calpastatin | 2,32 | 0,39 |
| 201049_s_at | RPS18 | ribosomal protein S18 | 2,32 | 0,06 |
| 221666_s_at | ASC | apoptosis-associated speck-like protein containing a CARD | 2,31 | 0,10 |
| 200933_x_at | RPS4X | ribosomal protein S4, X-linked | 2,31 | 0,06 |
| 201924_at | MLLT2 | myeloid/lymphoid or mixed-lineage leukemia (trithorax homolog, Drosophila); translocated to, 2 | 2,31 | 0,10 |
| 209143_s_at | CLNS1A | chloride channel, nucleotide-sensitive, 1A | 2,31 | 0,06 |
| 202130_at | RIOK3 | RIO kinase 3 (yeast) | 2,31 | 0,06 |
| 208881_x_at | IDI1 | isopentenyl-diphosphate delta isomerase | 2,31 | 0,26 |
| 202910_s_at | CD97 | CD97 antigen | 2,30 | 0,06 |
| 209189_at | FOS | v-fos FBJ murine osteosarcoma viral oncogene homolog | 2,30 | 0,06 |
| 200716_x_at | RPL13A | ribosomal protein L13a | 2,30 | 0,06 |
| 200818_at | ATP5O | ATP synthase, H+ transporting, mitochondrial F1 complex, O subunit (oligomycin sensitivity conferring protein) | 2,30 | 0,14 |
| 205090_s_at | NAGPA | N-acetylglucosamine-1-phosphodiester alpha-N-acetylglucosaminidase | 2,30 | 0,06 |
| 205668_at | LY75 | lymphocyte antigen 75 | 2,30 | 1,25 |
| 215171_s_at | TIMM17A | translocase of inner mitochondrial membrane 17 homolog A (yeast) | 2,30 | 0,48 |
| 204646_at | DPYD | dihydropyrimidine dehydrogenase | 2,30 | 0,32 |
| 201176_s_at | ARCN1 | archain 1 | 2,30 | 0,17 |
| 200082_s_at | RPS7 | ribosomal protein S7 | 2,29 | 0,06 |
| 201953_at | CIB1 | calcium and integrin binding 1 (calmyrin) | 2,29 | 0,06 |
| 213738_s_at | ATP5A1 | ATP synthase, H+ transporting, mitochondrial F1 complex, alpha subunit, isoform 1, cardiac muscle | 2,29 | 0,06 |
| 203676_at | GNS | glucosamine (N-acetyl)-6-sulfatase (Sanfilippo disease IIID) | 2,29 | 0,46 |
| 202061_s_at | SEL1L | sel-1 suppressor of lin-12-like (C. elegans) | 2,29 | 0,06 |
| 205633_s_at | ALAS1 | aminolevulinate, delta-, synthase 1 | 2,29 | 0,06 |
| 201322_at | ATP5B | ATP synthase, H+ transporting, mitochondrial F1 complex, beta polypeptide | 2,29 | 0,06 |
| 200092_s_at | --- | --- | 2,28 | 0,06 |
| 209630_s_at | FBXW2 | F-box and WD-40 domain protein 2 | 2,28 | 0,06 |
| 201128_s_at | ACLY | ATP citrate lyase | 2,28 | 0,84 |
| 200022_at | RPL18 | ribosomal protein L18 | 2,28 | 0,06 |
| 218150_at | ARL5 | ADP-ribosylation factor-like 5 | 2,28 | 0,06 |
| 205174_s_at | QPCT | glutaminyl-peptide cyclotransferase (glutaminyl cyclase) | 2,27 | 0,06 |
| 201534_s_at | UBL3 | ubiquitin-like 3 | 2,27 | 0,06 |
| 208623_s_at | VIL2 | villin 2 (ezrin) | 2,27 | 0,06 |
| 200936_at | RPL8 | ribosomal protein L8 | 2,27 | 0,06 |
| 201360_at | CST3 | cystatin C (amyloid angiopathy and cerebral hemorrhage) | 2,27 | 0,17 |
| 219547_at | COX15 | COX15 homolog, cytochrome c oxidase assembly protein (yeast) | 2,27 | 0,06 |
| 202314_at | CYP51A1 | cytochrome P450, family 51, subfamily A, polypeptide 1 | 2,27 | 0,14 |
| 217792_at | SNX5 | sorting nexin 5 | 2,27 | 0,06 |
| 202447_at | DECR1 | 2,4-dienoyl CoA reductase 1, mitochondrial | 2,27 | 0,20 |
| 217945_at | BTBD1 | BTB (POZ) domain containing 1 | 2,26 | 0,14 |
| 200027_at | NARS | asparaginyl-tRNA synthetase | 2,26 | 0,32 |
| 200807_s_at | HSPD1 | heat shock 60kDa protein 1 (chaperonin) | 2,26 | 0,17 |
| 220741_s_at | PPA2 | inorganic pyrophosphatase 2 | 2,26 | 0,06 |
| 205480_s_at | UGP2 | UDP-glucose pyrophosphorylase 2 | 2,26 | 0,10 |
| 200945_s_at | KIAA0905 | yeast Sec31p homolog | 2,26 | 0,20 |
| 202562_s_at | C14orf1 | chromosome 14 open reading frame 1 | 2,26 | 0,06 |
| 207233_s_at | MITF | microphthalmia-associated transcription factor | 2,26 | 0,06 |
| 201201_at | CSTB | cystatin B (stefin B) | 2,25 | 0,06 |
| 201256_at | COX7A2L | cytochrome c oxidase subunit VIIa polypeptide 2 like | 2,25 | 0,06 |
| 200613_at | AP2M1 | adaptor-related protein complex 2, mu 1 subunit | 2,25 | 0,10 |
| 201592_at | EIF3S3 | eukaryotic translation initiation factor 3, subunit 3 gamma, 40kDa | 2,25 | 0,06 |
| 221059_s_at | CHST6 | carbohydrate (N-acetylglucosamine 6-O) sulfotransferase 6 | 2,24 | 0,06 |
| 208855_s_at | STK24 | serine/threonine kinase 24 (STE20 homolog, yeast) | 2,24 | 0,06 |
| 206854_s_at | MAP3K7 | mitogen-activated protein kinase kinase kinase 7 | 2,24 | 0,17 |
| 202442_at | AP3S1 | adaptor-related protein complex 3, sigma 1 subunit | 2,24 | 0,06 |
| 201816_s_at | GBAS | glioblastoma amplified sequence | 2,24 | 0,06 |
| 217747_s_at | RPS9 | ribosomal protein S9 | 2,24 | 0,06 |
| 200091_s_at | RPS25 | ribosomal protein S25 | 2,23 | 0,06 |
| 202769_at | CCNG2 | cyclin G2 | 2,23 | 0,06 |
| 200918_s_at | SRPR | signal recognition particle receptor ('docking protein') | 2,23 | 0,06 |
| 200845_s_at | PRDX6 | peroxiredoxin 6 | 2,23 | 0,06 |
| 212652_s_at | SNX4 | sorting nexin 4 | 2,22 | 0,17 |
| 207507_s_at | ATP5G3 | ATP synthase, H+ transporting, mitochondrial F0 complex, subunit c (subunit 9) isoform 3 | 2,22 | 0,06 |
| 200888_s_at | RPL23 | ribosomal protein L23 | 2,22 | 0,06 |
| 202090_s_at | UQCR | ubiquinol-cytochrome c reductase (6.4kD) subunit | 2,22 | 0,06 |
| 202722_s_at | GFPT1 | glutamine-fructose-6-phosphate transaminase 1 | 2,22 | 0,32 |
| 200975_at | PPT1 | palmitoyl-protein thioesterase 1 (ceroid-lipofuscinosis, neuronal 1, infantile) | 2,22 | 0,06 |
| 201129_at | SFRS7 | splicing factor, arginine/serine-rich 7, 35kDa | 2,22 | 0,61 |
| 201433_s_at | PTDSS1 | phosphatidylserine synthase 1 | 2,22 | 0,20 |
| 218313_s_at | GALNT7 | UDP-N-acetyl-alpha-D-galactosamine:polypeptide N-acetylgalactosaminyltransferase 7 (GalNAc-T7) | 2,21 | 0,26 |
| 201089_at | ATP6V1B2 | ATPase, H+ transporting, lysosomal 56/58kDa, V1 subunit B, isoform 2 | 2,21 | 0,46 |
| 210719_s_at | HMG20B | high-mobility group 20B | 2,21 | 0,06 |
| 208852_s_at | CANX | calnexin | 2,21 | 0,39 |
| 208885_at | LCP1 | lymphocyte cytosolic protein 1 (L-plastin) | 2,21 | 0,10 |
| 200025_s_at | RPL27 | ribosomal protein L27 | 2,20 | 0,06 |
| 202302_s_at | FLJ11021 | similar to splicing factor, arginine/serine-rich 4 | 2,20 | 0,06 |
| 205000_at | DDX3Y | DEAD (Asp-Glu-Ala-Asp) box polypeptide 3, Y-linked | 2,20 | 0,06 |
| 212120_at | PIGF | phosphatidylinositol glycan, class F | 2,19 | 0,20 |
| 209316_s_at | HBS1L | HBS1-like (S. cerevisiae) | 2,19 | 0,06 |
| 213735_s_at | COX5B | cytochrome c oxidase subunit Vb | 2,19 | 0,06 |
| 208768_x_at | RPL22 | ribosomal protein L22 | 2,19 | 0,06 |
| 201757_at | NDUFS5 | NADH dehydrogenase (ubiquinone) Fe-S protein 5, 15kDa (NADH-coenzyme Q reductase) | 2,19 | 0,06 |
| 213313_at | GAPCENA | rab6 GTPase activating protein (GAP and centrosome-associated) | 2,19 | 0,70 |
| 204401_at | KCNN4 | potassium intermediate/small conductance calcium-activated channel, subfamily N, member 4 | 2,19 | 0,06 |
| 210908_s_at | PFDN5 | prefoldin 5 | 2,19 | 0,06 |
| 207674_at | FCAR | Fc fragment of IgA, receptor for | 2,19 | 0,06 |
| 200708_at | GOT2 | glutamic-oxaloacetic transaminase 2, mitochondrial (aspartate aminotransferase 2) | 2,19 | 0,06 |
| 208692_at | RPS3 | ribosomal protein S3 | 2,19 | 0,06 |
| 202731_at | PDCD4 | programmed cell death 4 (neoplastic transformation inhibitor) | 2,19 | 0,10 |
| 203414_at | MMD | monocyte to macrophage differentiation-associated | 2,18 | 0,06 |
| 204744_s_at | IARS | isoleucine-tRNA synthetase | 2,18 | 0,32 |
| 200665_s_at | SPARC | secreted protein, acidic, cysteine-rich (osteonectin) | 2,18 | 0,17 |
| 203911_at | RAP1GA1 | RAP1, GTPase activating protein 1 | 2,18 | 0,06 |
| 209190_s_at | DIAPH1 | diaphanous homolog 1 (Drosophila) | 2,18 | 0,17 |
| 208829_at | TAPBP | TAP binding protein (tapasin) | 2,18 | 0,70 |
| 201285_at | MKRN1 | makorin, ring finger protein, 1 | 2,18 | 0,06 |
| 217990_at | GMPR2 | guanosine monophosphate reductase 2 | 2,18 | 0,06 |
| 202325_s_at | ATP5J | ATP synthase, H+ transporting, mitochondrial F0 complex, subunit F6 | 2,18 | 0,17 |
| 200757_s_at | CALU | calumenin | 2,18 | 0,06 |
| 208909_at | UQCRFS1 | ubiquinol-cytochrome c reductase, Rieske iron-sulfur polypeptide 1 | 2,18 | 0,26 |
| 208720_s_at | RNPC2 | RNA-binding region (RNP1, RRM) containing 2 | 2,18 | 0,06 |
| 201791_s_at | DHCR7 | 7-dehydrocholesterol reductase | 2,18 | 0,70 |
| 203067_at | PDHX | pyruvate dehydrogenase complex, component X | 2,17 | 0,06 |
| 208905_at | CYCS | cytochrome c, somatic | 2,17 | 0,32 |
| 200030_s_at | SLC25A3 | solute carrier family 25 (mitochondrial carrier; phosphate carrier), member 3 | 2,17 | 0,06 |
| 200086_s_at | COX4I1 | cytochrome c oxidase subunit IV isoform 1 | 2,17 | 0,06 |
| 202003_s_at | ACAA2 | acetyl-Coenzyme A acyltransferase 2 (mitochondrial 3-oxoacyl-Coenzyme A thiolase) | 2,17 | 0,06 |
| 202236_s_at | SLC16A1 | solute carrier family 16 (monocarboxylic acid transporters), member 1 | 2,17 | 0,06 |
| 218217_at | RISC | likely homolog of rat and mouse retinoid-inducible serine carboxypeptidase | 2,17 | 0,06 |
| 218190_s_at | HSPC051 | ubiquinol-cytochrome c reductase complex (7.2 kD) | 2,16 | 0,10 |
| 201257_x_at | RPS3A | ribosomal protein S3A | 2,16 | 0,06 |
| 221619_s_at | MTCH1 | mitochondrial carrier homolog 1 (C. elegans) | 2,16 | 0,06 |
| 200710_at | ACADVL | acyl-Coenzyme A dehydrogenase, very long chain | 2,16 | 0,06 |
| 203039_s_at | NDUFS1 | NADH dehydrogenase (ubiquinone) Fe-S protein 1, 75kDa (NADH-coenzyme Q reductase) | 2,16 | 0,06 |
| 200797_s_at | MCL1 | myeloid cell leukemia sequence 1 (BCL2-related) | 2,15 | 0,06 |
| 220755_s_at | C6orf48 | chromosome 6 open reading frame 48 | 2,15 | 0,06 |
| 210949_s_at | EIF3S8 | eukaryotic translation initiation factor 3, subunit 8, 110kDa | 2,15 | 0,17 |
| 200061_s_at | RPS24 | ribosomal protein S24 | 2,15 | 0,06 |
| 201135_at | ECHS1 | enoyl Coenzyme A hydratase, short chain, 1, mitochondrial | 2,15 | 0,26 |
| 215111_s_at | TSC22 | transforming growth factor beta-stimulated protein TSC-22 | 2,15 | 0,26 |
| 201113_at | TUFM | Tu translation elongation factor, mitochondrial | 2,15 | 0,06 |
| 200018_at | RPS13 | ribosomal protein S13 | 2,15 | 0,06 |
| 200817_x_at | RPS10 | ribosomal protein S10 | 2,15 | 0,06 |
| 212694_s_at | PCCB | propionyl Coenzyme A carboxylase, beta polypeptide | 2,15 | 0,06 |
| 200955_at | IMMT | inner membrane protein, mitochondrial (mitofilin) | 2,15 | 0,06 |
| 202929_s_at | DDT | D-dopachrome tautomerase | 2,15 | 0,06 |
| 200603_at | PRKAR1A | protein kinase, cAMP-dependent, regulatory, type I, alpha (tissue specific extinguisher 1) | 2,14 | 0,14 |
| 210978_s_at | TAGLN2 | transgelin 2 | 2,14 | 0,06 |
| 200651_at | GNB2L1 | guanine nucleotide binding protein (G protein), beta polypeptide 2-like 1 | 2,14 | 0,06 |
| 201619_at | PRDX3 | peroxiredoxin 3 | 2,14 | 0,26 |
| 204041_at | MAOB | monoamine oxidase B | 2,14 | 0,06 |
| 200686_s_at | SFRS11 | splicing factor, arginine/serine-rich 11 | 2,14 | 0,17 |
| 201665_x_at | RPS17 | ribosomal protein S17 | 2,14 | 0,06 |
| 200903_s_at | AHCY | S-adenosylhomocysteine hydrolase | 2,14 | 0,32 |
| 210260_s_at | GG2-1 | TNF-induced protein | 2,14 | 0,84 |
| 201651_s_at | PACSIN2 | protein kinase C and casein kinase substrate in neurons 2 | 2,13 | 0,06 |
| 200674_s_at | RPL32 | ribosomal protein L32 | 2,13 | 0,06 |
| 204716_at | D10S170 | DNA segment on chromosome 10 (unique) 170 | 2,13 | 0,06 |
| 200002_at | RPL35 | ribosomal protein L35 | 2,13 | 0,06 |
| 211275_s_at | GYG | glycogenin | 2,13 | 0,48 |
| 202110_at | COX7B | cytochrome c oxidase subunit VIIb | 2,13 | 0,10 |
| 201443_s_at | ATP6AP2 | ATPase, H+ transporting, lysosomal accessory protein 2 | 2,13 | 0,10 |
| 201754_at | COX6C | cytochrome c oxidase subunit VIc | 2,13 | 0,06 |
| 202803_s_at | ITGB2 | integrin, beta 2 (antigen CD18 (p95), lymphocyte function-associated antigen 1; macrophage antigen 1 (mac-1) beta subunit) | 2,12 | 0,10 |
| 205627_at | CDA | cytidine deaminase | 2,12 | 0,10 |
| 220253_s_at | ST7 | suppression of tumorigenicity | 2,12 | 0,26 |
| 200629_at | WARS | tryptophanyl-tRNA synthetase | 2,12 | 0,46 |
| 208904_s_at | RPS28 | ribosomal protein S28 | 2,12 | 0,06 |
| 209089_at | RAB5A | RAB5A, member RAS oncogene family | 2,12 | 0,14 |
| 201403_s_at | MGST3 | microsomal glutathione S-transferase 3 | 2,12 | 0,10 |
| 203190_at | NDUFS8 | NADH dehydrogenase (ubiquinone) Fe-S protein 8, 23kDa (NADH-coenzyme Q reductase) | 2,12 | 0,17 |
| 211962_s_at | ZFP36L1 | zinc finger protein 36, C3H type-like 1 | 2,12 | 0,48 |
| 208370_s_at | DSCR1 | Down syndrome critical region gene 1 | 2,12 | 0,06 |
| 201938_at | CDK2AP1 | CDK2-associated protein 1 | 2,12 | 0,48 |
| 217874_at | SUCLG1 | succinate-CoA ligase, GDP-forming, alpha subunit | 2,12 | 0,06 |
| 208813_at | GOT1 | glutamic-oxaloacetic transaminase 1, soluble (aspartate aminotransferase 1) | 2,11 | 0,06 |
| 209139_s_at | PRKRA | protein kinase, interferon-inducible double stranded RNA dependent activator | 2,11 | 0,06 |
| 219161_s_at | CKLF | chemokine-like factor | 2,11 | 1,08 |
| 202845_s_at | RALBP1 | ralA binding protein 1 | 2,10 | 0,26 |
| 207785_s_at | RBPSUH | recombining binding protein suppressor of hairless (Drosophila) | 2,10 | 0,39 |
| 209046_s_at | GABARAPL2 | GABA(A) receptor-associated protein-like 2 | 2,10 | 0,14 |
| 210532_s_at | C14orf2 | chromosome 14 open reading frame 2 | 2,10 | 0,06 |
| 208787_at | MRPL3 | mitochondrial ribosomal protein L3 | 2,10 | 0,48 |
| 209102_s_at | HBP1 | HMG-box transcription factor 1 | 2,10 | 0,06 |
| 200026_at | RPL34 | ribosomal protein L34 | 2,10 | 0,06 |
| 221471_at | TDE1 | tumor differentially expressed 1 | 2,10 | 0,20 |
| 209095_at | DLD | dihydrolipoamide dehydrogenase (E3 component of pyruvate dehydrogenase complex, 2-oxo-glutarate complex, branched chain keto acid dehydrogenase complex) | 2,10 | 0,70 |
| 203104_at | CSF1R | colony stimulating factor 1 receptor, formerly McDonough feline sarcoma viral (v-fms) oncogene homolog | 2,10 | 0,46 |
| 217819_at | GCP16 | Golgi complex-associated protein of 16kDa | 2,10 | 0,20 |
| 205726_at | DIAPH2 | diaphanous homolog 2 (Drosophila) | 2,10 | 0,06 |
| 200877_at | CCT4 | chaperonin containing TCP1, subunit 4 (delta) | 2,09 | 0,14 |
| 204517_at | PPIC | peptidylprolyl isomerase C (cyclophilin C) | 2,09 | 0,06 |
| 210244_at | CAMP | cathelicidin antimicrobial peptide | 2,09 | 0,06 |
| 204847_at | ZNF-U69274 | zinc finger protein | 2,09 | 0,10 |
| 201568_at | QP-C | low molecular mass ubiquinone-binding protein (9.5kD) | 2,09 | 0,14 |
| 209606_at | PSCDBP | pleckstrin homology, Sec7 and coiled-coil domains, binding protein | 2,09 | 0,61 |
| 200869_at | RPL18A | ribosomal protein L18a | 2,09 | 0,06 |
| 201012_at | ANXA1 | annexin A1 | 2,09 | 0,06 |
| 208726_s_at | EIF2S2 | eukaryotic translation initiation factor 2, subunit 2 beta, 38kDa | 2,09 | 0,06 |
| 200912_s_at | EIF4A2 | eukaryotic translation initiation factor 4A, isoform 2 | 2,09 | 0,10 |
| 201336_at | VAMP3 | vesicle-associated membrane protein 3 (cellubrevin) | 2,09 | 0,06 |
| 201722_s_at | GALNT1 | UDP-N-acetyl-alpha-D-galactosamine:polypeptide N-acetylgalactosaminyltransferase 1 (GalNAc-T1) | 2,09 | 1,08 |
| 213225_at | PPM1B | protein phosphatase 1B (formerly 2C), magnesium-dependent, beta isoform | 2,08 | 0,10 |
| 209166_s_at | MAN2B1 | mannosidase, alpha, class 2B, member 1 | 2,08 | 0,06 |
| 202748_at | GBP2 | guanylate binding protein 2, interferon-inducible | 2,08 | 0,10 |
| 200011_s_at | ARF3 | ADP-ribosylation factor 3 | 2,08 | 0,70 |
| 201341_at | ENC1 | ectodermal-neural cortex (with BTB-like domain) | 2,08 | 0,06 |
| 201994_at | MORF4L2 | mortality factor 4 like 2 | 2,08 | 0,14 |
| 201565_s_at | ID2 | inhibitor of DNA binding 2, dominant negative helix-loop-helix protein | 2,08 | 0,10 |
| 200032_s_at | RPL9 | ribosomal protein L9 | 2,08 | 0,06 |
| 203282_at | GBE1 | glucan (1,4-alpha-), branching enzyme 1 (glycogen branching enzyme, Andersen disease, glycogen storage disease type IV) | 2,08 | 0,48 |
| 200909_s_at | RPLP2 | ribosomal protein, large P2 | 2,08 | 0,06 |
| 201694_s_at | EGR1 | early growth response 1 | 2,08 | 0,06 |
| 200793_s_at | ACO2 | aconitase 2, mitochondrial | 2,08 | 0,06 |
| 212205_at | H2AV | histone H2A.F/Z variant | 2,07 | 0,39 |
| 201094_at | RPS29 | ribosomal protein S29 | 2,07 | 0,06 |
| 208998_at | UCP2 | uncoupling protein 2 (mitochondrial, proton carrier) /// uncoupling protein 2 (mitochondrial, proton carrier) | 2,07 | 0,98 |
| 201390_s_at | CSNK2B | casein kinase 2, beta polypeptide | 2,07 | 0,06 |
| 203356_at | CAPN7 | calpain 7 | 2,07 | 0,06 |
| 202855_s_at | SLC16A3 | solute carrier family 16 (monocarboxylic acid transporters), member 3 | 2,07 | 0,20 |
| 206976_s_at | HSPH1 | heat shock 105kDa/110kDa protein 1 | 2,07 | 0,46 |
| 208631_s_at | HADHA | hydroxyacyl-Coenzyme A dehydrogenase/3-ketoacyl-Coenzyme A thiolase/enoyl-Coenzyme A hydratase (trifunctional protein), alpha subunit | 2,07 | 0,06 |
| 217080_s_at | HOMER2 | homer homolog 2 (Drosophila) | 2,07 | 0,06 |
| 214683_s_at | CLK1 | CDC-like kinase 1 | 2,06 | 1,08 |
| 203058_s_at | PAPSS2 | 3'-phosphoadenosine 5'-phosphosulfate synthase 2 | 2,06 | 0,06 |
| 202422_s_at | FACL4 | fatty-acid-Coenzyme A ligase, long-chain 4 | 2,06 | 0,10 |
| 202502_at | ACADM | acyl-Coenzyme A dehydrogenase, C-4 to C-12 straight chain | 2,06 | 0,26 |
| 202069_s_at | IDH3A | isocitrate dehydrogenase 3 (NAD+) alpha | 2,06 | 0,39 |
| 217938_s_at | KCMF1 | potassium channel modulatory factor 1 | 2,06 | 0,06 |
| 207038_at | SLC16A6 | solute carrier family 16 (monocarboxylic acid transporters), member 6 | 2,06 | 0,06 |
| 208828_at | POLE3 | polymerase (DNA directed), epsilon 3 (p17 subunit) | 2,06 | 0,20 |
| 212223_at | IDS | iduronate 2-sulfatase (Hunter syndrome) | 2,06 | 0,84 |
| 205756_s_at | F8 | coagulation factor VIII, procoagulant component (hemophilia A) | 2,05 | 0,06 |
| 221750_at | --- | Homo sapiens mRNA; cDNA DKFZp564P142 (from clone DKFZp564P142) | 2,05 | 1,46 |
| 206995_x_at | SCARF1 | scavenger receptor class F, member 1 | 2,05 | 0,06 |
| 208745_at | ATP5L | ATP synthase, H+ transporting, mitochondrial F0 complex, subunit g | 2,05 | 0,06 |
| 217983_s_at | RNASET2 | ribonuclease T2 | 2,05 | 0,06 |
| 202105_at | IGBP1 | immunoglobulin (CD79A) binding protein 1 | 2,04 | 0,06 |
| 216520_s_at | --- | --- | 2,04 | 0,06 |
| 205559_s_at | PCSK5 | proprotein convertase subtilisin/kexin type 5 | 2,04 | 0,06 |
| 202075_s_at | PLTP | phospholipid transfer protein | 2,04 | 0,14 |
| 221012_s_at | TRIM8 | tripartite motif-containing 8 /// tripartite motif-containing 8 | 2,04 | 0,06 |
| 200095_x_at | RPS10 | ribosomal protein S10 | 2,04 | 0,06 |
| 204348_s_at | AK3 | adenylate kinase 3 | 2,04 | 0,06 |
| 203739_at | ZNF217 | zinc finger protein 217 | 2,04 | 0,84 |
| 213385_at | CHN2 | chimerin (chimaerin) 2 | 2,04 | 0,39 |
| 205249_at | EGR2 | early growth response 2 (Krox-20 homolog, Drosophila) | 2,04 | 0,06 |
| 202026_at | SDHD | succinate dehydrogenase complex, subunit D, integral membrane protein | 2,04 | 0,06 |
| 201088_at | KPNA2 | karyopherin alpha 2 (RAG cohort 1, importin alpha 1) | 2,04 | 1,08 |
| 203182_s_at | SRPK2 | SFRS protein kinase 2 /// SFRS protein kinase 2 | 2,03 | 0,84 |
| 218241_at | GOLGA5 | golgi autoantigen, golgin subfamily a, 5 | 2,03 | 0,14 |
| 201740_at | NDUFS3 | NADH dehydrogenase (ubiquinone) Fe-S protein 3, 30kDa (NADH-coenzyme Q reductase) | 2,03 | 0,06 |
| 201832_s_at | VDP | vesicle docking protein p115 | 2,03 | 1,08 |
| 201225_s_at | SRRM1 | serine/arginine repetitive matrix 1 | 2,03 | 0,20 |
| 201494_at | PRCP | prolylcarboxypeptidase (angiotensinase C) | 2,03 | 0,20 |
| 201921_at | GNG10 | guanine nucleotide binding protein (G protein), gamma 10 | 2,03 | 0,32 |
| 215794_x_at | GLUD2 | glutamate dehydrogenase 2 | 2,03 | 0,06 |
| 203980_at | FABP4 | fatty acid binding protein 4, adipocyte | 2,03 | 0,06 |
| 200024_at | RPS5 | ribosomal protein S5 | 2,03 | 0,06 |
| 214581_x_at | TNFRSF21 | tumor necrosis factor receptor superfamily, member 21 | 2,03 | 0,06 |
| 221494_x_at | eIF3k | eukaryotic translation initiation factor 3 subunit k | 2,03 | 0,06 |
| 201034_at | ADD3 | adducin 3 (gamma) | 2,03 | 0,20 |
| 209043_at | PAPSS1 | 3'-phosphoadenosine 5'-phosphosulfate synthase 1 | 2,02 | 0,70 |
| 212038_s_at | VDAC1 | voltage-dependent anion channel 1 | 2,02 | 0,61 |
| 201885_s_at | DIA1 | diaphorase (NADH) (cytochrome b-5 reductase) | 2,02 | 0,06 |
| 208645_s_at | --- | Homo sapiens PRO2640 mRNA, complete cds | 2,02 | 0,06 |
| 204102_s_at | EEF2 | eukaryotic translation elongation factor 2 | 2,02 | 0,17 |
| 204039_at | CEBPA | CCAAT/enhancer binding protein (C/EBP), alpha | 2,02 | 0,20 |
| 200012_x_at | RPL21 | ribosomal protein L21 | 2,02 | 0,06 |
| 208130_s_at | TBXAS1 | thromboxane A synthase 1 (platelet, cytochrome P450, family 5, subfamily A) /// thromboxane A synthase 1 (platelet, cytochrome P450, family 5, subfamily A) | 2,02 | 0,26 |
| 202638_s_at | ICAM1 | intercellular adhesion molecule 1 (CD54), human rhinovirus receptor | 2,02 | 3,83 |
| 203538_at | CAMLG | calcium modulating ligand | 2,01 | 0,06 |
| 201738_at | GC20 | translation factor sui1 homolog | 2,01 | 0,32 |
| 200017_at | RPS27A | ribosomal protein S27a | 2,01 | 0,06 |
| 200031_s_at | RPS11 | ribosomal protein S11 | 2,01 | 0,06 |
| 200886_s_at | PGAM1 | phosphoglycerate mutase 1 (brain) | 2,01 | 0,06 |
| 202263_at | CYB5R1 | cytochrome b5 reductase 1 (B5R.1) | 2,01 | 0,06 |
| 201506_at | TGFBI | transforming growth factor, beta-induced, 68kDa | 2,01 | 0,48 |
| 201612_at | ALDH9A1 | aldehyde dehydrogenase 9 family, member A1 | 2,01 | 0,14 |
| 212321_at | SGPL1 | sphingosine-1-phosphate lyase 1 | 2,01 | 0,17 |
| 211939_x_at | BTF3 | basic transcription factor 3 | 2,01 | 0,06 |
| 201018_at | EIF1A | eukaryotic translation initiation factor 1A | 2,00 | 0,20 |
| 207016_s_at | ALDH1A2 | aldehyde dehydrogenase 1 family, member A2 | 2,00 | 0,70 |
